# Identification of microRNA editing sites in clear cell renal cell carcinoma

**DOI:** 10.1038/s41598-023-42302-y

**Published:** 2023-09-13

**Authors:** Yulong Liu, Shiyong Guo, Wenping Xie, Huaide Yang, Wanran Li, Nan Zhou, Jun Yang, Guangchen Zhou, Chunyi Mao, Yun Zheng

**Affiliations:** 1 State Key Laboratory of Primate Biomedical Research, Institute of Primate Translational Medicine, Kunming University of Science and Technology, Kunming, 650500 Yunnan China; 2 College of Landscape and Horticulture, Yunnan Agricultural University, Kunming, 650201 Yunnan China; 3 Faculty of Life Science and Technology, Kunming University of Science and Technology, Kunming, 650500 Yunnan China; 4 Faculty of Information Engineering and Automation, Kunming University of Science and Technology, Kunming, 650500 Yunnan China; 5 School of Criminal Investigation, Yunnan Police College, Kunming, 650223 Yunnan China

**Keywords:** Renal cell carcinoma, Renal cell carcinoma, Computational biology and bioinformatics

## Abstract

Clear cell renal cell carcinoma (ccRCC) is a malignant tumor originating from the renal tubular epithelium. Although the microRNAs (miRNAs) transcriptome of ccRCC has been extensively studied, the role of miRNAs editing in ccRCC is largely unknown. By analyzing small RNA sequencing profiles of renal tissues of 154 ccRCC patients and 22 normal controls, we identified 1025 miRNA editing sites from 246 pre-miRNAs. There were 122 editing events with significantly different editing levels in ccRCC compared to normal samples, which include two A-to-I editing events in the seed regions of *hsa-mir-376a-3p* and *hsa-mir-376c-3p*, respectively, and one C-to-U editing event in the seed region of *hsa-mir-29c-3p*. After comparing the targets of the original and edited miRNAs, we found that *hsa-mir-376a-1_49g*, *hsa-mir-376c_48g* and *hsa-mir-29c_59u* had many new targets, respectively. Many of these new targets were deregulated in ccRCC, which might be related to the different editing levels of *hsa-mir-376a-3p*, *hsa-mir-376c-3p*, *hsa-mir-29c-3p* in ccRCC compared to normal controls. Our study sheds new light on miRNA editing events and their potential biological functions in ccRCC.

## Introduction

Renal cell carcinoma (RCC), which is formed by malignant proliferation of tubular epithelial cells, is the most common malignant tumor of kidney^1^. According to the histological classification, there are many subtypes of RCC, among which clear cell renal cell carcinoma (ccRCC) is the most prevalent subtype, accounting for about 75% of all RCCs^1^. Although some ccRCC cases can be surgically resected, the metastatic rate of ccRCC is high, and about 30% of patients who present with metastases at first screening are not candidates for surgery^2,3^. Therefore, early detection and targeted therapy of ccRCC is the most effective way to reduce the number of deaths from this disease^4^.

MicroRNAs (miRNAs) are a class of single-stranded non-coding RNAs with approximately 22 nucleotides that can undergo extensive post-transcriptional modifications^5,6^. As important regulatory molecules in biological processes, miRNAs are involved in almost all cellular pathways and pathological processes, including cancer initiation, progression and metastasis^7^. Mature miRNAs pair with their target mRNAs through the seed regions (the first eight nucleotides from the 5${\prime }$ ends of miRNAs) to induce mRNA degradation or translational repression^8^. The regulation of gene expression by miRNAs is diverse and complex. A miRNA can bind to many different targets, and a target can be regulated by multiple miRNAs^9^. Therefore, even a single nucleotide change in a miRNA, especially if it occurs within the seed region, leads to severe changes of its targets and also affects the expression of its targets.

In ccRCC, miRNAs are reported as either oncomiRNAs or tumor suppressors^10^. For example, *hsa-miR-21-5p* was overexpressed in ccRCC tissues compared to normal controls and correlated with the downregulation of PPAR-$\alpha$ in ccRCC^11^. Ji et al. reported that *hsa-miR-155-5p* was upregulated in ccRCC, and inhibition of *hsa-miR-155-5p* could significantly suppress the proliferation, colony formation, migration and invasion and induce G1 phase arrest and apoptosis^12^. In addition, *hsa-miR-193a-3p* and *hsa-miR-224-3p* as oncogenic miRNAs^13^, activated the PI3K/Akt pathway and targeted glycosylation-related enzymes to mediate cell proliferation, migration and invasion in ccRCC. *hsa-miR-30a-5p*^14^ and *hsa-miR-30c-5p*^15^ were downregulated in ccRCC, and were associated with tumor aggressiveness^16^. Cui et al. found that *hsa-miR-99a-5p* was downregulated in ccRCC and correlated with overall survival^17^. Moreover, *hsa-miR-187-3p* presents lower levels in tumor tissue and plasma of patients with ccRCC, and lower levels of *hsa-miR-187-3p* were associated with higher tumor grade and stage^18^. In summary, a large number of miRNAs play crucial roles in the initiation and progression of ccRCC.

RNA editing is a prevalent and conserved post-transcriptional mechanism that plays a crucial role in the diversification of gene expression and transcriptome complexity across various organisms. This process involves specific proteins that catalyze chemical modifications to RNA molecules during their generation processes, leading to the alterations, deletions, and/or insertions of bases^19,20^. RNA is edited in a variety of ways. The most widely reported editing type is adenosine to inosine (A-to-I) editing, catalyzed by adenosine deaminase acting on RNA (ADAR) enzymes^19^. This is followed by editing of cytosine (C) to uracil (U), which is catalyzed by the apolipoprotein B mRNA editing catalytic polypeptide-like (APOBEC) proteins^19,20^. Furthermore, some RNAs are modified on their 3${\prime }$ ends to add additional nucleotides, such as A or U^21–23^.

It has been found that a large number of RNA editing events also exist in miRNAs^24–37^ and the A-to-I miRNA editing events in cancer have attracted some attention^38–40^. For example, *ADAR2* is responsible for editing *hsa-miR-21-3p/5p*, *hsa-miR-221-5p* and *hsa-miR-222-3p*, which exhibit high levels and are known to promote cancer progression in glioblastoma^41^. In a study on miRNA editing in pan-cancer, the edited *hsa-miR-200b-3p* promotes cell invasion and migration by targeting *LIFR*, a metastasis suppressor^39^. A-to-I edited *hsa-miR-589-3p* targets *ADAM12* and original *hsa-miR-589-3p* targets *PCDH9*, a tumor suppressor related to glioma progression^42^. A-to-I editing level of *hsa-miR-589-3p* is reduced in glioblastoma, which promotes the proliferation and invasion of brain cancer cells^42^. In addition, miRNA editing also plays an important role in neurological diseases. For example, A-to-I editing of *hsa-miR-497-5p* is enhanced in Parkinson’s disease (PD), which potentially promotes progressive neurodegeneration of PD patients^37^. In summary, miRNA editing is associated with the initiation and progression of numerous cancers. However, miRNA editing events in ccRCC are largely unknown, which has become a major obstacle and blind spot for the study of ccRCC.

In this study, to comprehensively characterize miRNA editing and/or modification sites in ccRCC, we systematically analyzed small RNA sequencing profiles of 154 ccRCC patients and 22 healthy controls. We identified 1025 miRNA mutation and/or editing (M/E) sites with significant editing levels in these samples. In addition, 122 M/E sites showed significantly different editing levels in ccRCC patients compared to control samples. Among them, we focused on 3 editing events that occurred in the seed regions and predicted the target genes of their corresponding edited miRNAs. We explored the potential functions of new target genes of edited miRNAs and examined expression patterns of these new targets in ccRCC using existing gene expression data. Our results suggested that A-to-I edited *hsa-mir-376a-1-3p* and *hsa-mir-376c-3p* targeted *BCAT1* and *MTHFD2*, respectively, and C-to-U edited *hsa-mir-29c-3p* targeted *RASSF8*. Presumably, because the A-to-I editing levels of *hsa-mir-376a-1-3p* and *hsa-mir-376c-3p* were reduced in ccRCC, *BCAT1* and *MTHFD2* were upregulated in ccRCC. Similarly, potentially due to increased C-to-U editing level of *hsa-mir-29c-3p*, *RASSF8* was significantly downregulated in ccRCC. These results suggest that miRNA editing contributes to initiation and progression of ccRCC.

## Results

### Summary of mutation and editing sites identified in miRNAs

**Figure 1:**
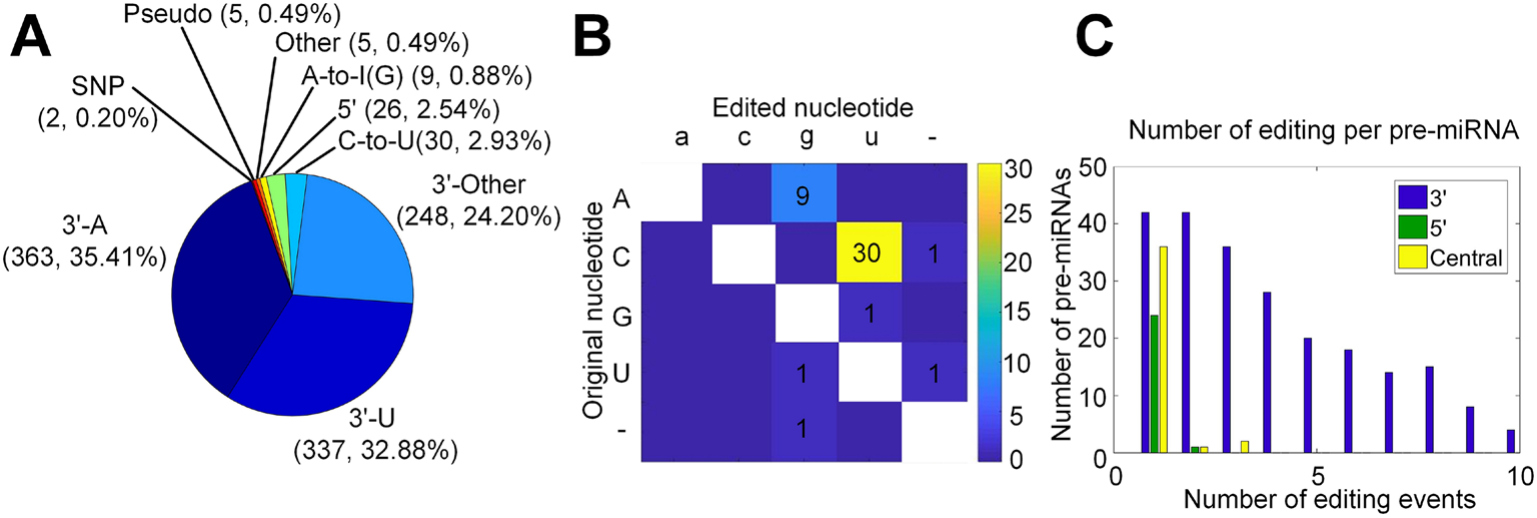
The number of significant M/E sites in miRNAs and their categories in the analyzed sRNA libraries. (**A**) The categories of significant M/E sites in miRNAs. (**B**) The editing sites and numbers in the central region of mature miRNAs. (**C**) The distribution of the numbers of pre-miRNAs with different numbers of $3^{\prime }$-, $5^{\prime }$-editing and Central editing sites.

The MiRME pipeline^43^ was used to identify miRNA M/E sites from the collected 176 sRNA-seq profiles. In total, 1025 M/E sites with significant editing levels were identified (as listed in Supplementary Table S2.1). As mentioned above, these M/E sites were divided into 9 categories (Fig. 1A and Supplementary Table S2.2). We found that there were more 3${\prime }$-addition sites than 5${\prime }$ editing sites (Fig.  1A and Supplementary Tables S2.3, S2.4). In addition, M/E sites in the central regions of mature miRNAs include 9 (0.88%) A-to-I, 30 (2.93%) C-to-U, 5 (2.54%) Other and 2 (0.20%) SNP sites.

We further investigated the miRNA editing events that occured in the central regions of mature miRNAs (Supplementary Table S2.5), i.e., A-to-I, C-to-U and Other. We counted the base changes of these three editing types (Fig. 1B), and found 9 A-to-I, 30 C-to-U, and 5 Other editing events. One insertion and two deletion events were also found (Fig. 1B). Furthermore, we also investigated the distributions of these events within different regions of mature miRNAs, including the 5${\prime }$ ends, 3${\prime }$ ends, and central regions. Our results indicated that 3${\prime }$-addition was the most common type of RNA editing events of miRNAs. As shown in Fig. 1C, the numbers of editing events at the 3${\prime }$ ends were significantly higher than those at the 5${\prime }$ ends and the central regions. For the vast majority of miRNAs, only 1 or 2 editing events happened at the 5${\prime }$ ends and the central regions.

### Nine miRNA A-to-I editing sites in ccRCC

**Figure 2:**
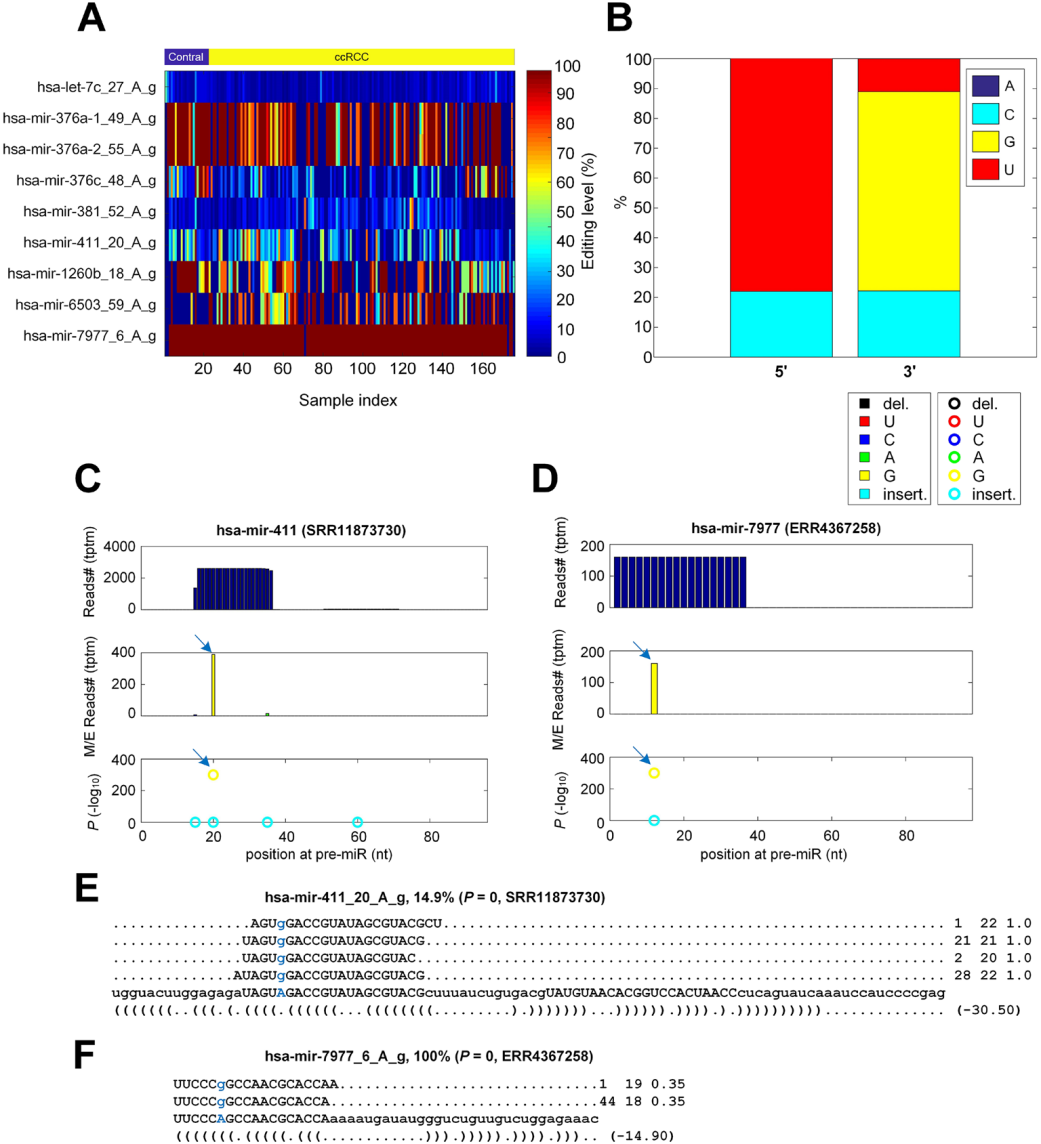
The details of 9 identified A-to-I editing sites in miRNAs. (**A**) The editing levels of the 9 A-to-I editing sites in the 176 selected sRNA profiles. (**B**) The percentages of nucleotides around of 9 A-to-I sites. (**C**) The MiRME map of *hsa-mir-411* in one of the ccRCC samples selected (SRR11873730). (**D**) The MiMRE map of *hsa-mir-7977* in one of the ccRCC samples selected (ERR4367258). (**E**) The details of *hsa-mir-411_20_A_g* in SRR11878730. (**F**) The details of *hsa-mir-7977_6_A_g* in ERR4367258. In Part (**C**) to (**D**), The three panels from top to bottom show the total number of reads (Tags Per Ten Million sequencing reads, tptm), the number of M/E reads with variations (tptm), and the *p*-values of M/E events, respectively. In Part (**E**) to (**F**), the blue nucleotides are original and edited. And the three numbers on the right indicate the frequency of the read, the length of the read, and the weight of the read at this locus, respectively.

Totally, 9 A-to-I editing sites with significant editing levels were identified from the 176 samples (Fig. 2A). The editing levels of *hsa-mir-376a-1* and *hsa-mir-376a-2* were almost identical, probably due to their high sequence similarity. We then counted the nucleotides around these 9 A-to-I editing sites, and found that the 5${\prime }$ and 3${\prime }$ ends of these A-to-I editing sites had clear U and G base preference, respectively (Fig. 2B), which was consistent with results in literature^27,29,43^. As examples, the detailed information of two A-to-I editing sites in *hsa-mir-411* and *hsa-mir-7977* in two ccRCC samples (SRR11873730 and ERR4367258, respectively) were presented in Fig. 2C and D, respectively. As shown in Fig. 2E and F, a large number of sequencing reads in these samples supported the two A-to-I editing sites.

### Thirty miRNA C-to-U editing sites in ccRCC

**Figure 3:**
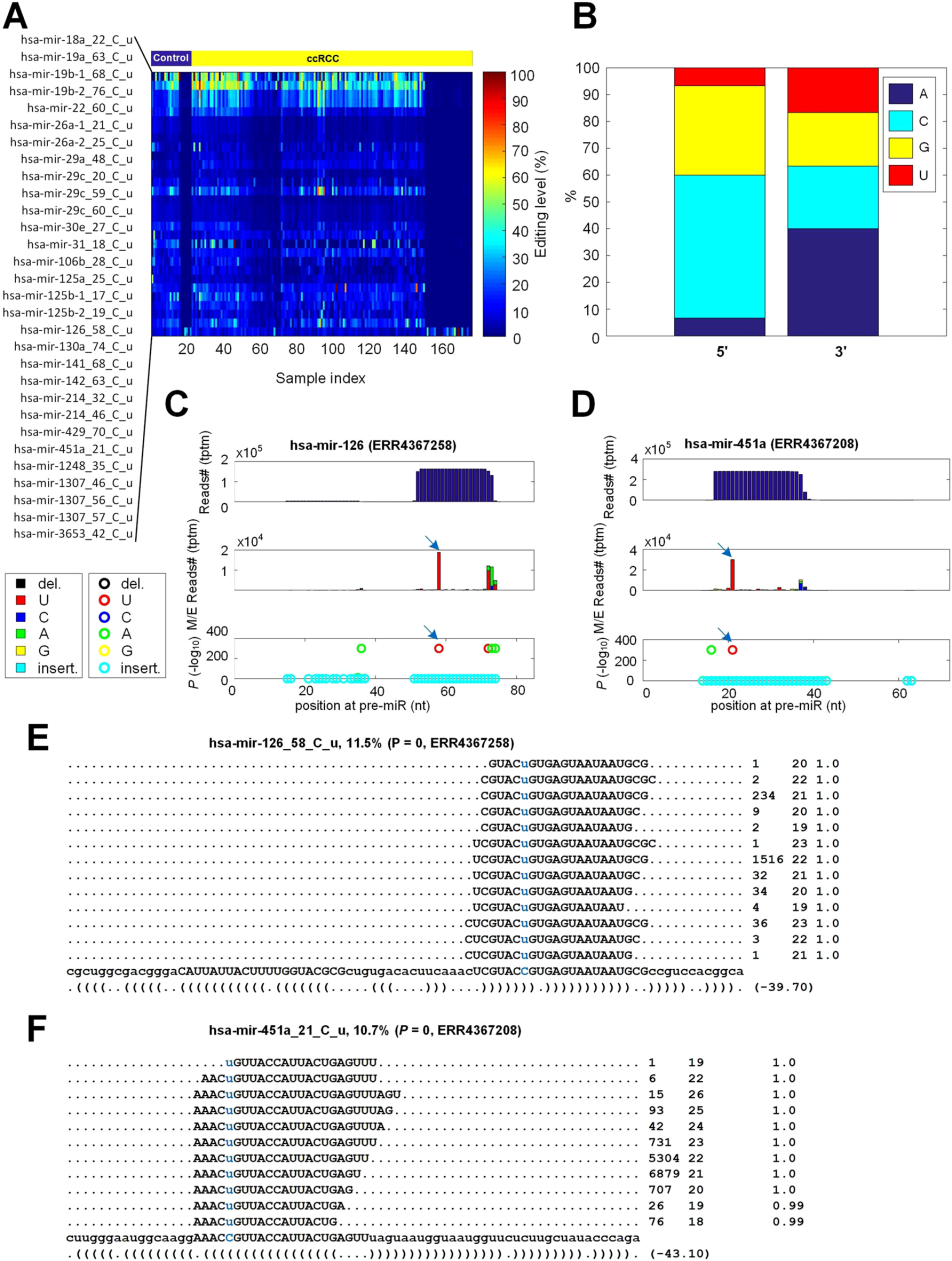
The details of 30 identified C-to-U editing sites in miRNAs. (**A**) The editing levels of the 30 C-to-U sites in the 176 selected sRNA-seq profiles. (**B**) The percentages of nucleotides around the 30 C-to-U sites. (**C**) The MiRME map of *hsa-mir-126* in one of the ccRCC samples selected (ERR4367258). (**D**) The MiRME map of *hsa-mir-451a* in one of the ccRCC samples selected (ERR4367208). (**E**) The details of *hsa-mir-126_58_C_u* in ERR4367258. (**F**) The details of *hsa-mir-451a_21_C_u* in ERR4367208. In Part (**C**) to (**D**), The three panels from top to bottom show the total number of reads (Tags Per Ten Million sequencing reads, tptm), the number of M/E reads with variations (tptm), and the *p*-values of M/E events, respectively. In Part (**E**) to (**F**), the blue nucleotides are original and edited. And the three numbers on the right indicate the frequency of the read, the length of the read, and the weight of the read at this locus, respectively.

We carefully studied the 30 C-to-U editing sites and found that their editing levels were generally low in the 176 sample (Fig. 3A). Furthermore, nucleotides on the 5${\prime }$ sides of these C-to-U editing sites were biased toward C, while those on the 3${\prime }$ sides were biased toward A (Fig. 3B). Examples of two C-to-U editing sites were shown in Fig. 3C and D, respectively. In addition, the detailed editing sites and editing levels of these two miRNAs were shown in Fig. 3E and F, respectively. These two C-to-U editing sites were supported by thousands of sequencing reads in the samples used, indicating good reliabilities of these two editing sites.

### Two miRNA SNP sites in ccRCC

The identified M/E sites were compared with SNPs in miRNAs previously reported^44^ and NCBI dbSNP (v151) according to the criteria mentioned in Materials and Methods. Two SNPs (rs11614913 and rs2155248) were shown in Supplementary Fig. S1 and Supplementary Table S2.6, and the editing levels of these two SNPs in one normal and one ccRCC sample, respectively, were shown in Supplementary Fig. S1A. As shown in Supplementary Fig. S1B and S1C, these SNPs were supported by many sequencing reads in SRR11873716 and SRR11873719, respectively. Additionally, the editing levels of these two sites reached 100% (Supplementary Fig. S1D and S1E), consistent to the fact that these sites were SNPs.

### 122 M/E sites with significantly different editing levels in ccRCC

**Figure 4:**
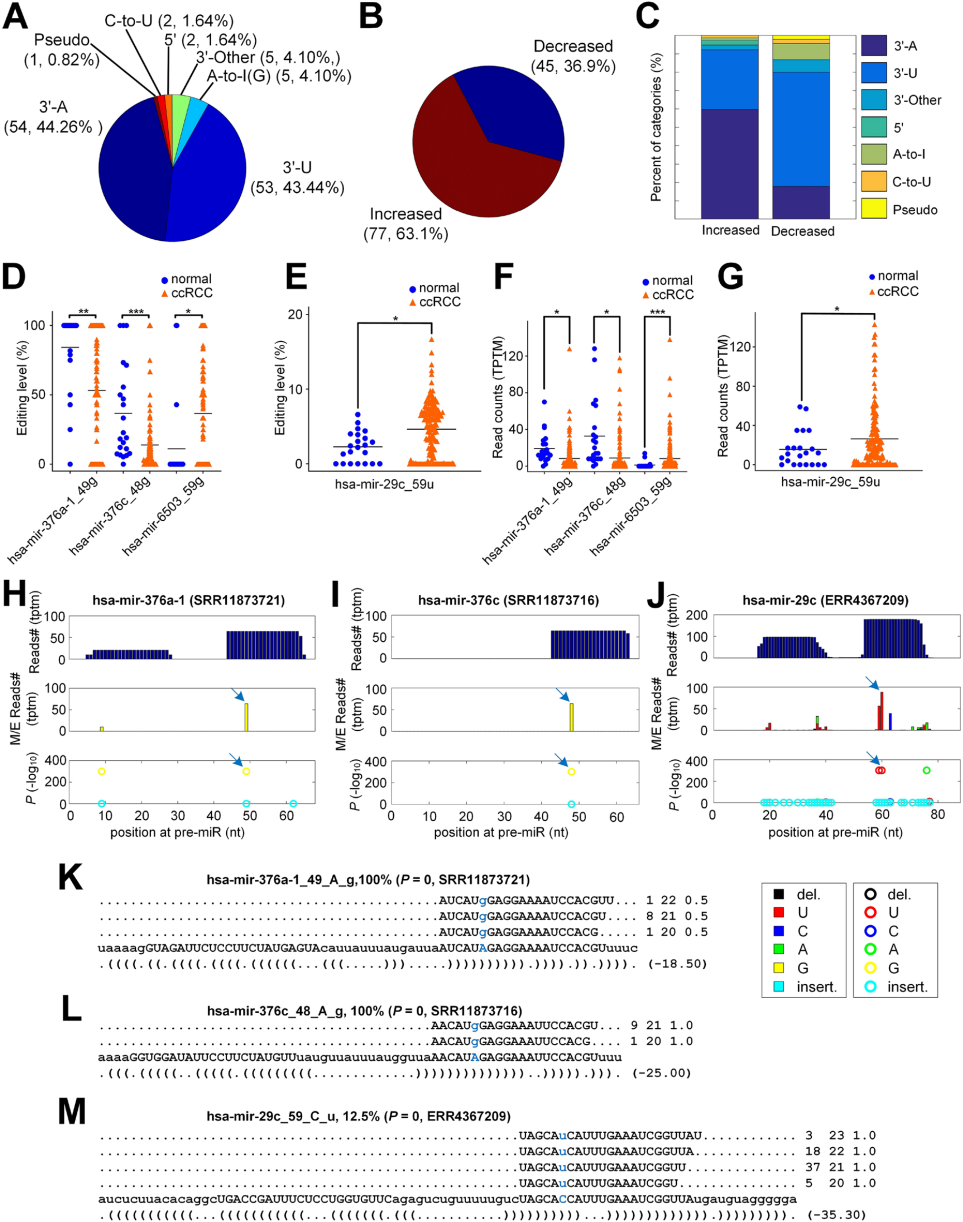
The 122 miRNA M/E sites that have significantly different editing levels in ccRCC samples when compared to normal samples. (**A**) The categories of the 122 M/E sites. (**B**) The change of editing levels of 122 M/E sites in ccRCC samples compared to normal samples. (**C**) The percentages of different categories of 122 M/E sites with significantly different editing levels in ccRCC samples. (**D**) Comparison of editing levels of *hsa-mir-376a-1_49g*, *hsa-mir-376c_48g* and *hsa-mir-6503_59g* in ccRCC and normal samples. (**E**) Comparison of editing levels of *hsa-mir-29c_59u* in ccRCC and normal samples. (**F**) Comparison of expression levels of *hsa-mir-376a-1_49g*, *hsa-mir-376c_48g* and *hsa-mir-6503_59g* in ccRCC and normal samples. (**G**) Comparison of expression levels of *hsa-mir-29c_59u* in ccRCC and normal samples. (**H**) The MiRME map of *hsa-mir-376a-1* in one of the ccRCC samples (SRR11873721). (**I**) The MiRME map of *hsa-mir-376c* in one of the normal samples (SRR11873716). (**J**) The MiRME map of *hsa-mir-29c* in one of the normal samples (ERR4367209). **(K)** The details of *hsa-mir-376a-1_49_A_g* in SRR11873721. (**L**) The details of *hsa-mir-376c_48_A_g* in SRR11873716. (**M**) The details of *hsa-mir-29c_59_C_u* in ERR4367209. In Part (**D**) to (**G**), “*”: $p <$ 0.05; “**”: $p <$ 0.01; “***”: $p <$ 0.001 and “NS”: not significant, i.e., $p >$ 0.05.

We obtained 122 miRNA M/E sites with significantly different editing levels in ccRCC samples (n = 154) compared to normal controls (n = 22) by using Mann-Whitney *U*-tests (corrected $p < 0.05$) (Supplementary Table S3). The 122 miRNA M/E sites were divided into 7 categories. 112 of these 122 sites were 3$^{\prime }$-addition events (3$^{\prime }$-A, 3$^{\prime }$-U, and 3$^{\prime }$-Other). In addition, there were 5 A-to-I editing sites, 2 C-to-U editing sites, and the remaining two were 5$^{\prime }$ end sites and one Pseudo site, i.e., potential false positive site (Fig. 4A). Most of the 122 M/E sites had increased editing levels (77 M/E sites, 63.1%) and 45 M/E sites (36.9%) had decreased editing levels in ccRCC samples when compared to normal controls (Fig. 4B). The categories of M/E sites with increased and decreased editing levels in ccRCC samples were shown in Fig. 4C. In M/E sites with increased editing levels in ccRCC, the 3${\prime }$-A editing events accounted for the largest portion, while the 3${\prime }$-U editing events accounted for the largest part in those with decreased editing levels in ccRCC compared to normal controls.

In ccRCC, the editing levels of *hsa-mir-376a-1_49_A_g* and *hsa-mir-376c_48_A_g* were significantly decreased (Fig. 4D), while those of *hsa-mir-6503_59_A_g* and *hsa-mir-29c_59_u* were significantly increased (Fig. 4D and E, respectively). We also investigated the levels of these edited miRNAs and found that *hsa-mir-376a-1_49g* and *hsa-mir-376c_48g* were significantly downregulated in ccRCC samples, while the levels of *hsa-mir-6503_59g* and *hsa-mir-29c_59u* were significantly upregulated in ccRCC (Fig. 4F and G, respectively). Figure 4H–M illustrated the details of the three edited miRNAs, *hsa-mir-376a-1_49_A_g*, *hsa-mir-376c_48_A_g* and *hsa-mir-29c_59_u* in one ccRCC sample (SRR11873721), one normal sample (SRR11873716) and one ccRCC sample (ERR4367209), respectively.

### The expression levels of *ADAR* and *APOBEC* genes in ccRCC

As shown in Supplementary Fig. S2 and Supplementary Table S7, the expression levels of the *ADAR* genes that catalyzed A-to-I editing of miRNAs were examined. In the TCGA data, *ADAR1* was downregulated in ccRCC compared to normal controls (Supplementary Fig. S2A) which was consistent with the decreased editing levels of *hsa-mir-376a-1_49_A_g*, *hsa-mir-376a-2_55_A_g*, and *hsa-mir-376c_48_A_g*. However, in the other two cohorts of gene expression profiles, *ADAR1* was upregulated, suggesting that more researches were needed to reveal the role of *ADAR1* in the editing of miRNAs in ccRCC. We found that *ADAR2* was significantly upregulated in ccRCC samples compared to normal controls in the three selected cohorts of gene expression datasets (Supplementary Fig. S2B). Meanwhile, the editing level of *hsa-mir-6503_59_A_g* was significantly increased in ccRCC samples compared to normal controls (Fig. 4D), suggesting that *ADAR2* mediated the editing of *hsa-mir-6503* in ccRCC and contributed to its increased editing levels in ccRCC compared to normal controls. *ADAR3* was downregulated in one cohort of gene expression profiles, and had no significant change in the other two datasets (Supplementary Fig. S2C).

The expression of the *APOBEC* genes that mediated C-to-U editing of miRNAs was also examined. As shown in Supplementary Fig. S3 and Supplementary Table S8, the expression levels of most *APOBEC* family members showed a significantly upward trend in ccRCC tumors, including *AICDA*, *APOBEC3A*, *APOBEC3B*, *APOBEC3C*, *APOBEC3D*,* APOBEC3F*, *APOBEC3G* and *APOBEC3H* genes (Supplementary Fig. S3A, D–J, respectively). In our results, the editing level of *hsa-mir-29c_59_C_u* was significantly increased in ccRCC samples (Fig. 4E). More researches are needed to clarify which *APOBEC* gene is the most promising enzyme that mediates the C-to-U editing of *hsa-mir-29c_59_C_u* and other miRNA C-to-U editing sites in ccRCC.

### The expression levels of * TENT* genes in ccRCC

Terminal nucleotidyltransferases (*TENTs*) modify RNA at post-transcriptional level, which regulates the stability and activity of RNA^45^. TENT2 and TENT4B proteins catalyze 3${\prime }$ end adenylation of miRNAs^21,46–50^. TUT4 and TUT7 proteins are terminal uridine transferases that belong to the TENT3 subfamily, which have the ability to catalyze the 3${\prime }$ uridylation of miRNAs^23,51–53^. The levels of these *TENT* genes in ccRCC and normal controls were examined (Supplementary Table S9). In ccRCC, the level of *TENT2* was significantly higher (Supplementary Fig. S4A–C), while *TENT4B* was significantly downregulated (Supplementary Fig. S4A and C). There are more 3${\prime }$-A sites with increased editing levels in ccRCC than 3${\prime }$-A sites with decreased editing levels in ccRCC (Fig. 4C), suggesting that *TENT2* might be the major enzyme for the 3${\prime }$-A editing of miRNAs in ccRCC, while the downregulation of *TENT4B* could be responsible for the decreased 3${\prime }$-A editing levels of some miRNAs in ccRCC.

The levels of *TUT4* had no significantly different expression in ccRCC compared to normal controls (Supplementary Fig. S4A to C), but the expression of *TUT7* was significantly upregulated in ccRCC (Supplementary Fig. S4A–C). These results suggest that some 3${\prime }$-U editing sites of miRNAs with increased editing levels in ccRCC (Fig. 4C, left column) are mainly catalyzed by TUT7.

### Identification of targets for A-to-I and C-to-U edited miRNAs

Three M/E sites (*hsa-mir-376a-1_49_A_g* and *hsa-mir-376c_48_A_g* and *hsa-mir-29c_59_C_u*) in seed regions of mature miRNAs were selected for subsequent analysis, because these sites had significantly different editing levels in ccRCC and the levels of their corresponding edited miRNAs were also significantly different and in the same directions of changes when compared their editing levels in ccRCC with normal controls.

**Figure 5:**
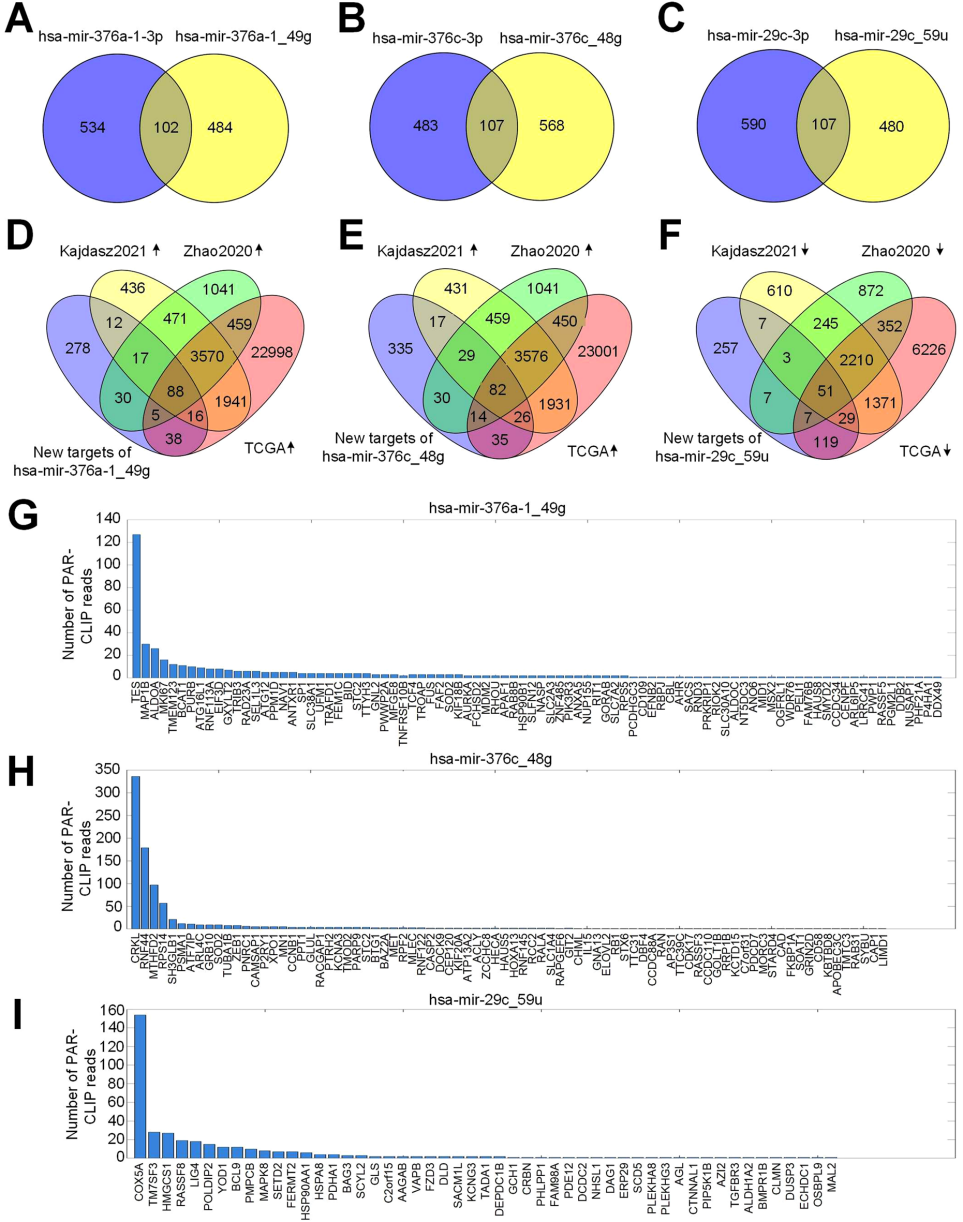
Comparisons of targets of edited miRNAs and deregulated genes in clear cell renal cell carcinoma. Venn diagrams were prepared with Venny (https://bioinfogp.cnb.csic.es/tools/venny/). (**A**–**C**) The venn diagrams depict the targets of the original and edited *hsa-mir-376a-1*, *hsa-mir-376c* and *hsa-mir-29c*, respectively. (**D**) The overlap between the new target genes of *hsa-mir-376a-1_49g* and genes upregulated in ccRCC in three gene expression datasets. (**E**) The overlap between the new target genes of *hsa-mir-376c_48g* and genes upregulated in ccRCC in three gene expression datasets. (**F**) The overlap between the new target genes of *hsa-mir-29c_59u* and genes downregulated in ccRCC in three gene expression datasets. (**G**)–(**I**) The numbers of PAR-CLIP reads for the 88, 82 and 51 overlapped targets of *hsa-mir-376a-1_49g*, *hsa-mir-376c_48g*, and *hsa-mir-29c_59u* in Part (**D**) to (**F**), respectively.

To decrease false positives and increase the reliabilities of predicted miRNA targets, we used 11 PAR-CLIP sequencing profiles. The principle of PAR-CLIP (Photoactivatable Ribonucleoside-Enhanced Crosslinking and Immunoprecipitation) technology is used to detect the crosslinking of RNA-binding proteins (RBPs) to RNA molecules, followed by immunoprecipitation and sequencing to identify the binding sites^54^. Through PAR-CLIP experiments, the interaction between RISC (including AGO proteins and miRNAs) and mRNA can be identified^54^. The sequences of the miRNA binding sites were obtained through PAR-CLIP experiments (targeting one of the AGO proteins) and then were sequenced^54^. Next, the MiCPAR algorithm^55^ was used to identify the targets of original and edited miRNAs by analyzing 11 PAR-CLIP profiles of AGO proteins (see Methods for details). We found that the original and edited *hsa-mir-376a-1-3p* had 102 common target genes, and 484 new target genes of *hsa-mir-376a-1_49g* were found (Fig. 5A and Supplementary Tables S4.1–S4.3). The original and edited *hsa-mir-376c-3p* had 107 common target genes, and *hsa-mir-376c_48g* had 568 new target genes (Fig. 5B and Supplementary Tables S5.1–S5.3). The original and edited *hsa-mir-29c-3p* had 107 identical target genes, and *hsa-mir-29c_59u* had 480 new target genes (Fig. 5C and Supplementary Tables S6.1–S6.3). Next, we compared the new target genes of *hsa-mir-376a-1_49g*, *hsa-mir-376c_48g* and *hsa-mir-29c_59u* to deregulated genes in ccRCC. We found that in the three gene expression datasets selected (GSE151419, GSE126964 and TCGA), 88 and 82 target genes of *hsa-mir-376a-1_49g* and *hsa-mir-376c_48g* were commonly significantly upregulated in ccRCC (Fig. 5D,E and Supplementary Tables S4.4, S5.4), respectively. Additionally, 51 target genes of *hsa-mir-29c_59u* were commonly significantly downregulated in ccRCC (Fig. 5F and Supplementary Table S6.4).

The numbers of PAR-CLIP reads for the 88, 82 and 51 overlapped target genes in Fig. 5D–F were shown in Fig. 5G–I, respectively. Then, among these target genes of *hsa-mir-376a-1_49g*, *hsa-mir-376c_48g*, and *hsa-mir-29c_59u*, genes with more than 10 PAR-CLIP reads with T-to-C variations were retained for further analysis (Supplementary Tables S4.5, S5.5, S6.5, respectively). Among the retained target genes of *hsa-mir-376a-1_49g*, *hsa-mir-376c_48g* and *hsa-mir-29c_59u*, *BCAT1*, *MTHFD2* and *RASSF8* were further analyzed after examining their functional relevance in ccRCC by reading their literature, respectively.

**Figure 6:**
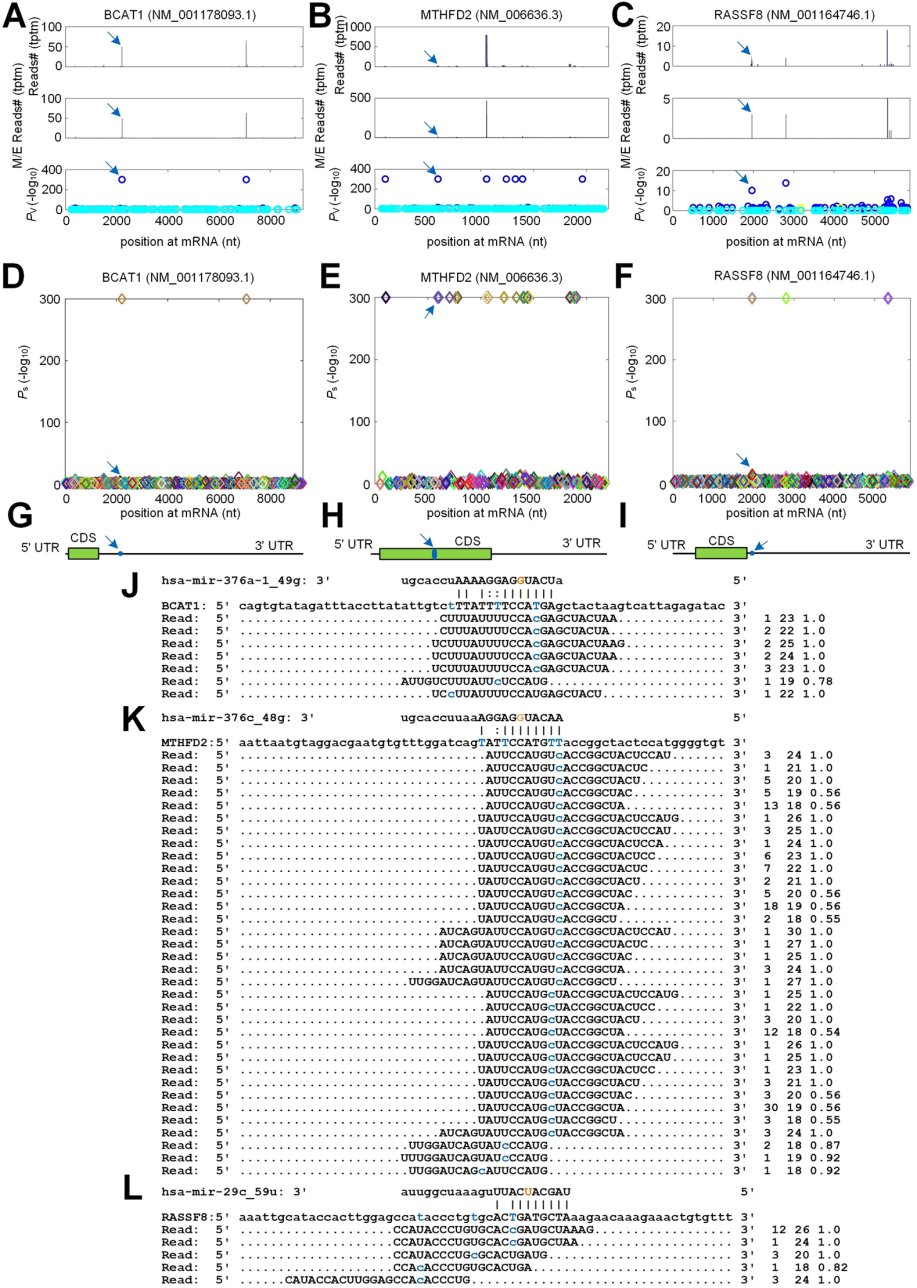
The details of the selected target genes of edited ***hsa-mir-376a-3p***, ***hsa-mir-376c-3p*** **and** ***hsa-mir-29c-3p***. (**A**–**C**) The distributions of PAR-CLIP reads on *BCAT1* (NM_001178093.1), *MTHFD2* (NM_006636.3) and *RASSF8* (NM_001164746.1), respectively. The peaks pointed by blue arrows were the complementary sites of *hsa-mir-376a-1_49g*, *hsa-mir-376c_48g* and *hsa-mir-29c_59u*. (**D**–**F**) The identified miRNA sites and their *P*$_{s}$ values on *BCAT1* (NM_001178093.1), *MTHFD2* (NM_006636.3) and *RASSF8* (NM_001164746.1), respectively. (**G**–**I**) The loci of the complementary sites of *hsa-mir-376a-1_49g*, *hsa-mir-376c_48g* and *hsa-mir-29c_59u* on *BCAT1* (NM_001178093.1), *MTHFD2* (NM_006636.3) and *RASSF8* (NM_001164746.1), respectively. (**J**–**L**) The details of complementary sites of *hsa-mir-376a-1_49g*, *hsa-mir-376c_48g* and *hsa-mir-29c_59u* on *BCAT1* (NM_001178093.1), *MTHFD2* (NM_006636.3) and *RASSF8* (NM_001164746.1), respectively, as well as PAR-CLIP reads on these sites. In Part (**J**) to (**L**), blue nucleotides indicate T-to-C on mRNA and PAR-CLIP sequencing reads. The orange nucleotides in the seed regions of *hsa-mir-376a-1_49g*, *hsa-mir-376c_48g*, and *hsa-mir-29c_59u* indicate the Guanine and Uracil introduced by the RNA editing. And the three numbers on the right indicate the number of raw sequencing read, the length of the read, and the weight of the read at this locus, respectively. In Parts (**A**), (**D**) and (**G**), the locus pointed by blue arrow is the complementary site of *hsa-mir-376a-1_49g* on *BCAT1* (NM_001178093.1) in Part (**J**), and the same relations apply for Parts (**B**), (**E**), (**H**) and (**K**), and Parts (**C**), (**F**), (**I**) and (**L**), respectively.

Figure 6A–C showed the distribution of PAR-CLIP reads for *BCAT1*,* MTHFD2* and* RASSF8*, respectively. These results indicated that PAR-CLIP reads were significantly accumulated at the complementary sites of *hsa-mir-376a-1_49g*, *hsa-mir-376c_48g* and *hsa-mir-29c_59u*, respectively. The identified miRNA complementary sites and their *P*$_{s}$ values on *BCAT1*, *MTHFD2* and *RASSF8* were shown in Fig. 6D–F, respectively. The complementary sites of *hsa-mir-376a-1_49g*, *hsa-mir-376c_48g* and *hsa-mir-29c_59u* were located in the 3$^{\prime }$UTR, CDS and 3$^{\prime }$UTR of *BCAT1*, *MTHFD2* and *RASSF8*, respectively (as shown in Fig. 6G to 6I, respectively). *BCAT1* and *MTHFD2* were upregulated in ccRCC, while *RASSF8* was downregulated in ccRCC (Supplementary Fig. S5 and Supplementary Tables S10.1–S10.3). Figure 6J–L showed the details of complementary sites of *hsa-mir-376a-1_49g*, *hsa-mir-376c_48g* and *hsa-mir-29c_59u* on *BCAT1* (NM_001178093.1), *MTHFD2* (NM_006636.3) and *RASSF8* (NM_001164746.1), respectively, as well as the PAR-CLIP reads at these sites.

### Functional analysis of A-to-I and C-to-U edited miRNAs

We performed Gene Ontology (GO) and KEGG pathway enrichment analysis to investigate the functions of the new target genes that were commonly deregulated in the three gene expression datasets selected for *hsa-mir-376a-1_49g*, *hsa-mir-376c_48g*, and *hsa-mir-29c_59u*, respectively (Supplementary Fig. S6, Supplementary Tables S11.1–S11.3 and S12.1–S12.3). The target genes of *hsa-mir-376a-1_49g* were enriched in many cancer-related pathways, including “Transcriptional misregulation in cancer”, “Small cell lung cancer”, “Prostate cancer”, “Pathways in cancer”, “p53 signaling pathway”, “Non-small cell lung cancer”, “Melanoma”, “Glioma”, “Endometrial cance”, “Colorectal cancer”, “Chronic myeloid leukemia” and “Breast cancer” (Supplementary Fig. S6A). The target genes of *hsa-mir-376c_48g* were mainly enriched in the “Pathways in cancer” and the “Rap1 signaling pathway” (Supplementary Fig. S6C). Additionally, “Metabolic pathways” was the most enriched pathway of the 51 target genes of *hsa-mir-29c_59u* (Supplementary Fig. S6E). These results suggest that *hsa-mir-376a-1_49g* and *hsa-mir-376c_48g* are directly related to ccRCC by targeting genes in many cancer-related pathways, while *hsa-mir-29c_59u* seems to be involved in the deregulation of genes in metabolic pathways.

## Discussion

We identified 1025 M/E sites with significant editing levels by analyzing 176 sRNA-seq profiles of ccRCC patients (n = 154) and healthy controls (n = 22). The M/E sites at the 3$^{\prime }$ end accounted for the majority, which was consistent with previous work^37,40,43,56–59^. We identified 122 editing events with significantly different editing levels in ccRCC samples compared to healthy control samples. The editing levels of 63.1% and 36.9% of these 122 miRNA editing events were increased and decreased in ccRCC patients, respectively, which may be related to the pathogenesis of ccRCC.

Subsequently, we focused on A-to-I and C-to-U miRNA editing events that occurred in the seed regions and were dysregulated in ccRCC. The editing levels of *hsa-mir-376a-1_49g* and *hsa-mir-376c_48g* were significantly lower in ccRCC than those in normal samples. In addition, their levels also showed the same significant downregulation in ccRCC samples compared to normal controls. The editing level of *hsa-mir-29c_59_C_u* and level of *hsa-mir-29c_59u* showed significant increases in ccRCC compared to normal controls. We compared the targets of original *hsa-miR-376a-3p*, *hsa-miR-376c-3p* and *hsa-miR-29c-3p* with the targets of edited miRNAs, *hsa-mir-376a-1_49g*, *hsa-mir-376c_48g * and *hsa-mir-29c_59u*. We found that *hsa-mir-376a-1_49g*, *hsa-mir-376c_48g* and *hsa-mir-29c_59u* could target 484, 568 and 480 additional novel targets, respectively.

C-to-U editing of miRNA was not widely reported. Interestingly, we detected 30 C-to-U editing events, much more than A-to-I editing events identified, in ccRCC. The more prevalence of C-to-U miRNA editing in ccRCC may be related to the high levels of *APOBEC* gene family in ccRCC (Supplementary Fig. S4). For examples, *APOBEC3C*, *APOBEC3F* and *APOBEC3G* were highly expressed in ccRCC samples compared to normal controls (as shown in Supplementary Figs. S4F, H and I, respectively). The fifth position of *hsa-miR-100-5p* was specifically edited in Treg^60^. C-to-U edited *hsa-miR-100-5p* repressed a new target *SMAD2*, which reduced the fraction of Treg in peripheral blood mononuclear cells^60^. The C-to-U editing of *hsa-mir-29c_59_C_u* in ccRCC might represent another case of functional C-to-U editing sites in miRNAs, because its editing levels significantly increased in ccRCC and the C-to-U edited *hsa-mir-29c-3p* potentially repressed *RASSF8* in ccRCC. As 11 of these 30 C-to-U editing sites were in seed regions (Fig. 3 and Supplementary Table S2.5), more of these C-to-U editing might be functional in tissue- or time-specific manners.

In our study, *BCAT1* and *MTHFD2* were upregulated in ccRCC and identified as target genes of *hsa-mir-376a-1_49g* and *hsa-mir-376c_48g* in ccRCC, respectively. *BCAT1* is overexpressed in many cancers and has been proposed as a marker for predicting cancer prognosis^61^. For example, overexpression of *BCAT1* promotes tumor growth in renal clear cell carcinoma^62^, lung cancer^63^, glioma^64^, ovarian cancer^65^, breast cancer^66^, hepatocellular carcinoma^67,68^, myeloid leukemia^69^, gastric cancer^70,71^, endometrial cancer^72^, non-small cell lung cancer^73^ and pan-cancer^74^. Moreover, *BCAT1* and its metabolites participate in the metabolism of cancer cells through different mechanisms. For example, the PI3K/AKT/mTOR signaling pathway is activated by *BCAT1* to promote the proliferation and angiogenesis of gastric cancer cells in vitro^70^. At the same time, in the study of lung adenocarcinoma, *BCAT1* promotes lung adenocarcinoma progression by enhancing mitochondrial function and NF-$\kappa$B pathway^75^.

Recent reports have shown that *MTHFD2* is also highly expressed in many cancers, including renal clear cell carcinoma^76,77^, pancreatic cancer,^78^ breast cancer^79^, myeloid leukemia^80^, non-small cell lung cancer^81,82^, hepatocellular carcinoma^83^, colorectal cancer^84^, breast cancer^85,86^, ovarian cancer^87^ , bladder cancer^88^, glioblastoma^89^, nasopharyngeal carcinoma^90^, oral squamous cell carcinoma^91^, and pan-cancer^92^. In addition, studies have reported the role of *MTHFD2* in tumor immune evasion. *MTHFD2* is induced by IFN-$\gamma$ and promotes basal and IFN-$\gamma$-induced PD-L1 expression. Mechanistically, *MTHFD2* drives folate circulation to maintain intracellular UDP-GlcNAc and cMYC O-GlcNAcylation, which promotes PD-L1 transcription^93^. Huang et al. found that *MTHFD2* promoted breast cancer cell proliferation through AKT signaling pathway, and high level of *MTHFD2* reduced the survival rate of breast cancer patients^79^.

In summary, the upregulation of *BCAT1* and* MTHFD2* in ccRCC may be due to the lower levels of *hsa-mir-376a_49g* and *hsa-mir-376c_48g* in ccRCC, respectively, which may contribute to the initiation and/or progression of ccRCC. Reduced A-to-I editing level of miRNAs were also noticed previously^38,39^. The editing levels of *hsa-mir-376a-1_49_A_g* and/or *hsa-mir-376c_48_A_g* were also reduced in many other cancers, such as glioblastoma^94^ and lung cancer^95^. The increased expression of *BCAT1* and *MFTHD2* might be due to the reduced editing levels of *hsa-mir-376a-1_49_A_g* and *hsa-mir-376c_48_A_g* in many cancers. Furthermore, it has been shown that the higher the editing level of *hsa-mir-376c_48_A_g* site in ccRCC, the better the prognosis and the longer the survival time of patients^38^. Therefore, reduced editing levels of *hsa-mir-376a-1_49_A_g* and *hsa-mir-376c_48_A_g* might represent a general mechanism in the initiation and/or progression of different cancers.

*RASSF8* has been reported to be a tumor suppressor gene with lower expression in gastric cancer^96,97^, colorectal cancer^98,99^, cervical cancer^100^, ovarian cancer^101^, melanoma^102^, esophageal squamous cell carcinoma^103^, lung cancer^104,105^ and osteosarcoma^106^. In addition, *RASSF8* is a potential therapeutic target for the prevention of many cancers^97,100,103,105^. In this study, *RASSF8*, the target gene of *hsa-mir-29c_59u*, was downregulated in ccRCC. Therefore, *RASSF8* may also play similar tumor suppressor function in ccRCC, and the higher level of *hsa-mir-29c_59u* in ccRCC may lead to the decreased expression of *RASSF8* in ccRCC, which consequently promotes the initiation and/or progression of ccRCC.

As shown in Fig. 5G–I, some genes with many PAR-CLIP reads were identified as targets of *hsa-mir-376a-1_49g* and *hsa-mir-376c_48g*, and *hsa-mir-29c_59u*, respectively. Although there currently are limited evidences about their functions in ccRCC, our results suggest that these genes represent promising directions for revealing the mechanisms of ccRCC in the future.

In summary, we presented a comprehensive view of miRNA editing and/or modification events in ccRCC, which promoted our understanding of miRNA editing and/or modification events in ccRCC. More researches are needed in the future to clarify the functional roles of different miRNA editing patterns in the pathogenesis of ccRCC. Our results also provided new clues for the clinical treatment of ccRCC, such as to promote the editing levels of *hsa-mir-376a_49_A_g* and *hsa-mir-376c_48_A_g* and to repress the editing level of *hsa-mir-29c_59_C_u*.

## Methods

### Small RNA-Seq data used

We collected 176 small RNA-Seq (sRNA-seq) profiles of ccRCC patients and healthy controls, including 154 samples of cancer tissue and 22 normal kidney tissue samples. These 176 sRNA-seq profiles were obtained from previous studies^107,108^, and their accession numbers were shown in Supplementary Table S1.1. The qualities of these sRNA-seq sequence data were evaluated with FastQC (v0.11.9)^109^.

### Genome and annotation of miRNAs used

The human genome sequence used was GRCh38, and downloaded from the UCSC Genome Browser^110^. The pri-miRNA sequences in GFF3 format and mature miRNA annotation files were derived from miRBase (v21)^111^.

### Identification of mutation and editing sites in miRNAs

Totally, 176 sRNA-seq profiles were analyzed using the MiRME pipeline with default settings^43^. Briefly, the raw reads were checked to retain the qualified reads with the sequencing scores greater than 30 for the first 25 nucleotides. Then, reads of at least 18 nt were retained after removal of the 3${\prime }$ adapters. BLASTN^112^ was used to compare the retained reads with the pre-miRNAs and the reads mapped to pre-miRNAs were retrieved. These reads, which were mapped to pre-miRNAs, were then aligned with the genome using Bowtie (v1.0.0)^113^. Then, the cross-mapping correction algorithm^28^ was used to examine the alignment of reads to the genome to calculate the weights or percentages of reads at different genomic loci. Results from different samples were then combined using separate programs in the MiRME package^43,55^. According to the location of M/E sites in miRNAs and mutations status of dbSNP, the M/E sites were divided into nine different editing types, i.e., A-to-I, C-to-U, 3$^{\prime }$-A, 3$^{\prime }$-U, 3$^{\prime }$-Other, 5$^{\prime }$-editing, Other, SNP and Pseudo^43^.

The identified M/E site was named with the pre-miRNA name, M/E site position in pre-miRNA, original nucleotide in upper case, the edited/mutated nucleotide in lower case. For example, *hsa-mir-376c_48_A_g* means an A-to-I editing at the 48th nucleotide of *hsa-mir-376c*. And edited miRNA was named by the pre-miRNA name, the M/E site position in pre-miRNA, and the edited/mutated nucleotide in lower case. For example, *hsa-mir-376c_48g * is the A-to-I edited *miR-376c-3p*.

The criteria for defining M/E sites with significant editing levels were as follows: (i) the relative levels of editing were at least 5%; (ii) editing events were supported by at least 10 reads; (iii) the threshold for sequencing reads score was 30; (iv) multiple test corrected *p*-values (with the Benjamini and Hochberg method^114^) were smaller than 0.05.

In order to remove M/E sites due to random sequencing errors, 1025 M/E sites that had significant editing levels in at least 10% of the 176 samples (18 samples) used in this study were retained for further analysis. The identified M/E sites were compared with known human miRNA editing sites, including the DARNED database^115^, the RADAR database^116^ and relevant literature^27,29,34,43,95,117,118^. Finally, A-to-I, C-to-U, and Other predicted M/E sites were manually checked.

### Identification of SNPs sites

The identified M/E sites were compared with the dbSNP (v151) database^119^ and previously reported SNPs in miRNAs^44^. An M/E site was considered as SNP if it satisfied the following criteria: (i) the genomic position of M/E site and SNP was identical, (ii) the original and edited nucleotides had the same nucleotides as the SNP’s allele, (iii) the M/E site had editing level of 100% in at least one of the 176 samples selected, and (iv) the M/E site occurred in the center of the miRNA.

### Identification of M/E sites with significantly different editing levels in ccRCC

The Mann-Whitney *U*-tests were used to analyze the differences between the editing levels of 1025 miRNA M/E sites in ccRCC (n = 154) and normal samples (n = 22). The *p*-values were corrected with the Benjamini-Hochberg correction method^114^. The M/E sites with corrected *p*-values smaller than 0.05 were defined as having significantly different editing levels in ccRCC.

DESeq2^120^ was used to analyze different expression levels (TPTM) of edited miRNAs between ccRCC and normal control samples. The miRNAs with corrected *p*-values smaller than 0.05 were defined as having different expression levels in ccRCC and normal control samples.

### Identification of targets for original and edited miRNAs

Among the editing sites with significant differences in editing levels between ccRCC tumors and normal tissues, a total of five editing events occurred in seed regions of mature miRNAs. We chose two A-to-I editing sites (*hsa-mir-376a-1_49_A_g* and *hsa-mir-376c_48_A_g*) and one C-to-U editing site (*hsa-mir-29c_59_C_u*), because their editing levels and the levels of their corresponding edited miRNAs had significant differences in ccRCC tumor tissues compared to normal controls. Then, we predicted the targets of original and edited miRNAs using the MiCPAR algorithm^55^. As shown in Supplementary Table S1.2, 11 PAR-CLIP sequences were used in the identification of miRNA targets and were downloaded from the NCBI SRA database. Seven PAR-CLIP profiles of HEK293 cell lines stably expressing FLAG/HA-tagged AGO1, AGO2, AGO3, and AGO4 proteins were reported by^54^. Another study included four PAR-CLIP profiles derived from HEK293 cell lines stably expressing HIS/FLAG/HA-tagged AGO1 and AGO2 proteins^121^. In order to obtain reads of acceptable quality, the raw reads of these 11 PAR-CLIP profiles were filtered to keep reads with sequencing scores of at least 30 for their first 25 nucleotides from 5${\prime }$ ends. The qualified reads were combined and the targets of miRNA were identified by the MiCPAR algorithm. The targets with at least one PAR-CLIP read with T-to-C variation were retained for further analysis.

### Analyzing expression of targets of edited miRNAs

In order to understand the expression of targets of edited miRNAs in ccRCC tumor tissues, we identified genes that were significantly differentially expressed in ccRCC samples compared to normal samples. We collected three batches of gene expression profiles of ccRCC and controls (Supplementary Table S1.3), of which two batches were obtained from^107,122^. Another set of gene expression profiles was obtained from TCGA (https://portal.gdc.cancer.gov/). The edgeR (v3.34.1) package^123^ was used for differential analysis of target genes. The glmFit and glmLRT functions in edgeR were used to build generalized linear models and to perform likelihood ratio tests, respectively. Genes with corrected *p*-values smaller than 0.05 were defined as having significantly different expression levels in ccRCC.

### Functional analysis of new target genes of edited miRNAs

We kept targets with at least one PAR-CLIP read with T-to-C variation. Then, the targets of original and edited miRNAs were compared to obtain the new targets of edited miRNAs. For edited miRNAs with increased editing levels in ccRCC, we compared their targets with genes that were downregulated in ccRCC because miRNAs normally repressed their target genes, and vice versa. We also analyzed the numbers of PAR-CLIP reads for the targets of edited miRNAs. We manually examined the functional relevance of some selected targets by reading the literature of the genes in the NCBI Gene database. The targets with more PAR-CLIP reads and functional relevance in ccRCC were preferred in our analysis.

KOBAS3.0^124^ was used to perform enrichment analysis of GO terms and KEGG pathways for new target genes of edited miRNAs. Significantly enriched GO terms and KEGG pathways were filtered with multiple test corrected *p*-values smaller than 0.05.

### Supplementary Information

Supplementary Information 1.

Supplementary Information 2.

Supplementary Information 3.

Supplementary Information 4.

Supplementary Information 5.

Supplementary Information 6.

Supplementary Information 7.

Supplementary Information 8.

Supplementary Information 9.

Supplementary Information 10.

Supplementary Information 11.

Supplementary Information 12.

## Data Availability

The 176 sRNA-seq sequencing profiles and 11 PAR-CLIP sequencing profiles were obtained from NCBI SRA database, and 3 gene expression profiles were obtained from NCBI GEO and TCGA database with accession numbers shown in Table S1.

## References

[1] Hsieh JJ (2017). Renal cell carcinoma. Nat. Rev. Dis. Primers..

[2] Choueiri TK, Motzer RJ (2017). Systemic therapy for metastatic renal-cell carcinoma. N. Engl. J. Med..

[3] Motzer RJ (2017). Kidney cancer, version 2.2017, NCCN clinical practice guidelines in oncology. J. Natl. Compr. Canc. Netw..

[4] Lai Y (2021). The tumour microenvironment and metabolism in renal cell carcinoma targeted or immune therapy. J. Cell. Physiol..

[5] Thomson JM (2006). Extensive post-transcriptional regulation of microRNAs and its implications for cancer. Genes Dev..

[6] Kim VN, Han J, Siomi MC (2009). Biogenesis of small RNAs in animals. Nat. Rev. Mol. Cell Biol..

[7] Hussen BM (2021). MicroRNA: A signature for cancer progression. Biomed. Pharmacother..

[8] Bartel DP (2009). MicroRNAs: Target recognition and regulatory functions. Cell.

[9] Ha M, Kim VN (2014). Regulation of microRNA biogenesis. Nat. Rev. Mol. Cell Biol..

[10] Braga EA, Fridman MV, Loginov VI, Dmitriev AA, Morozov SG (2019). Molecular mechanisms in clear cell renal cell carcinoma: Role of miRNAs and hypermethylated miRNA genes in crucial oncogenic pathways and processes. Front. Genet..

[11] Goujon M (2022). A double-negative feedback interaction between miR-21 and PPAR-$$\alpha$$ in clear renal cell carcinoma. Cancers.

[12] Ji H (2017). Overexpression of miR-155 in clear-cell renal cell carcinoma and its oncogenic effect through targeting FOXO3a. Exp. Ther. Med..

[13] Pan Y (2018). MiR-193a-3p and miR-224 mediate renal cell carcinoma progression by targeting alpha-2, 3-sialyltransferase IV and the phosphatidylinositol 3 kinase/Akt pathway. Mol. Carcinog..

[14] Ren Y, Zhang L, Zhang W, Gao Y (2021). MiR-30a suppresses clear cell renal cell carcinoma proliferation and metastasis by targeting LRP6. Hum. Cell.

[15] Huang J (2013). Hypoxia-induced downregulation of miR-30c promotes epithelial-mesenchymal transition in human renal cell carcinoma. Cancer Sci..

[16] Outeiro-Pinho G (2022). Epigenetically-regulated miR-30a/c-5p directly target TWF1 and hamper ccRCC cell aggressiveness. Transl. Res..

[17] Cui L (2012). MicroRNA-99a induces G1-phase cell cycle arrest and suppresses tumorigenicity in renal cell carcinoma. BMC Cancer.

[18] Zhao J (2013). MicroRNA-187, down-regulated in clear cell renal cell carcinoma and associated with lower survival, inhibits cell growth and migration though targeting B7–H3. Biochem. Biophys. Res. Commun..

[19] Brennicke A, Marchfelder A, Binder S (1999). RNA editing. FEMS Microbiol. Rev..

[20] Christofi T, Zaravinos A (2019). RNA editing in the forefront of epitranscriptomics and human health. J. Transl. Med..

[21] Burroughs AM (2010). A comprehensive survey of 3’ animal miRNA modification events and a possible role for 3’ adenylation in modulating miRNA targeting effectiveness. Genome Res..

[22] Kim Y-K, Heo I, Kim VN (2010). Modifications of small RNAs and their associated proteins. Cell.

[23] Gutiérrez-Vázquez C (2017). 3’ Uridylation controls mature microRNA turnover during CD4 T-cell activation. RNA.

[24] Luciano DJ, Mirsky H, Vendetti NJ, Maas S (2004). RNA editing of a miRNA precursor. RNA.

[25] Blow MJ (2006). RNA editing of human microRNAs. Genome Biol..

[26] Landgraf P (2007). A mammalian microRNA expression atlas based on small RNA library sequencing. Cell.

[27] Kawahara Y (2008). Frequency and fate of microRNA editing in human brain. Nucleic Acids Res..

[28] de Hoon MJ (2010). Cross-mapping and the identification of editing sites in mature microRNAs in high-throughput sequencing libraries. Genome Res..

[29] Alon S (2012). Systematic identification of edited microRNAs in the human brain. Genome Res..

[30] Ekdahl Y, Farahani HS, Behm M, Lagergren J, Öhman M (2012). A-to-I editing of microRNAs in the mammalian brain increases during development. Genome Res..

[31] Mingardi J, Musazzi L, De Petro G, Barbon A (2018). miRNA editing: New insights into the fast control of gene expression in health and disease. Mol. Neurobiol..

[32] Li L (2018). The landscape of miRNA editing in animals and its impact on miRNA biogenesis and targeting. Genome Res..

[33] Marceca GP (2021). Detecting and characterizing A-To-I microRNA editing in cancer. Cancers.

[34] Nishikura K (2016). A-to-I editing of coding and non-coding RNAs by ADARs. Nat. Rev. Mol. Cell Biol..

[35] Nigita, G. *et al.* ncRNA editing: Functional characterization and computational resources. *Computational Biology of Non-Coding RNA* 133–174 (2019). 10.1007/978-1-4939-8982-9_6 30635893

[36] Wang Y, Liang H (2018). When MicroRNAs meet RNA editing in cancer: A nucleotide change can make a difference. BioEssays.

[37] Lu C (2022). Characterizing relevant microRNA editing sites in Parkinson’s disease. Cells.

[38] Pinto Y, Buchumenski I, Levanon EY, Eisenberg E (2018). Human cancer tissues exhibit reduced A-to-I editing of miRNAs coupled with elevated editing of their targets. Nucleic Acids Res..

[39] Wang Y (2017). Systematic characterization of A-to-I RNA editing hotspots in microRNAs across human cancers. Genome Res..

[40] Xie W (2022). Identification of microRNA editing sites in three subtypes of leukemia. Front. Mol. Biosci..

[41] Tomaselli S (2015). Modulation of microRNA editing, expression and processing by ADAR2 deaminase in glioblastoma. Genome Biol..

[42] Cesarini V (2018). ADAR2/miR-589-3p axis controls glioblastoma cell migration/invasion. Nucleic Acids Res..

[43] Zheng Y (2016). Accurate detection for a wide range of mutation and editing sites of microRNAs from small RNA high-throughput sequencing profiles. Nucleic Acids Res..

[44] Han M, Zheng Y (2013). Comprehensive analysis of single nucleotide polymorphisms in human microRNAs. PLoS ONE.

[45] Yu S, Kim VN (2020). A tale of non-canonical tails: gene regulation by post-transcriptional RNA tailing. Nat. Rev. Mol. Cell Biol..

[46] Katoh T (2009). Selective stabilization of mammalian microRNAs by 3’ adenylation mediated by the cytoplasmic poly (A) polymerase GLD-2. Genes Dev..

[47] D’Ambrogio A, Gu W, Udagawa T, Mello CC, Richter JD (2012). Specific miRNA stabilization by Gld2-catalyzed monoadenylation. Cell Rep..

[48] Lee M (2014). Adenylation of maternally inherited microRNAs by wispy. Mol. Cell.

[49] Boele J (2014). PAPD5-mediated 3’ adenylation and subsequent degradation of miR-21 is disrupted in proliferative disease. Proc. Natl. Acad. Sci..

[50] Shukla S, Bjerke GA, Muhlrad D, Yi R, Parker R (2019). The RNase PARN controls the levels of specific miRNAs that contribute to p53 regulation. Mol. Cell.

[51] Jones MR (2009). Zcchc11-dependent uridylation of microRNA directs cytokine expression. Nat. Cell Biol..

[52] Jones MR (2012). Zcchc11 uridylates mature miRNAs to enhance neonatal IGF-1 expression, growth, and survival. PLoS Genet..

[53] Yang A (2019). 3’ uridylation confers miRNAs with non-canonical target repertoires. Mol. Cell.

[54] Hafner M (2010). Transcriptome-wide identification of RNA-binding protein and microRNA target sites by PAR-CLIP. Cell.

[55] Zheng Y (2018). Computational Non-coding RNA Biology.

[56] Zheng Y, Li T, Ren R, Shi D, Wang S (2014). Revealing editing and SNPs of microRNAs in colon tissues by analyzing high-throughput sequencing profiles of small RNAs. BMC Genom..

[57] Wang Q (2019). Identifying microRNAs and their editing sites in Macaca mulatta. Cells.

[58] Guo S (2022). MicroRNA editing patterns in Huntington’s disease. Sci. Rep..

[59] Wu X (2023). Characterizing microRNA editing and mutation sites in Autism Spectrum Disorder. Front. Mol. Neurosci..

[60] Negi V (2015). Altered expression and editing of miRNA-100 regulates iTreg differentiation. Nucleic Acids Res..

[61] Ananieva EA, Wilkinson AC (2018). Branched-chain amino acid metabolism in cancer. Curr. Opin. Clin. Nutr. Metab. Care.

[62] Huang W (2020). Bioinformatic gene analysis for possible biomarkers and therapeutic targets of hypertension-related renal cell carcinoma. Transl. Androl. Urol..

[63] Mao L (2021). Proteomic analysis of lung cancer cells reveals a critical role of BCAT1 in cancer cell metastasis. Theranostics.

[64] Tönjes M (2013). BCAT1 promotes cell proliferation through amino acid catabolism in gliomas carrying wild-type IDH1. Nat. Med..

[65] Wang Z-Q (2015). BCAT1 expression associates with ovarian cancer progression: Possible implications in altered disease metabolism. Oncotarget.

[66] Zhang L, Han J (2017). Branched-chain amino acid transaminase 1 (BCAT1) promotes the growth of breast cancer cells through improving mtor-mediated mitochondrial biogenesis and function. Biochem. Biophys. Res. Commun..

[67] Zheng Y-H (2016). BCAT1, a key prognostic predictor of hepatocellular carcinoma, promotes cell proliferation and induces chemoresistance to cisplatin. Liver Int..

[68] Ding, Y. *et al.* BCAT1, as a prognostic factor for HCC, can promote the development of liver cancer through activation of the AKT signaling pathway and EMT. *J. Mol. Histol.* 1–15 (2022). 10.1007/s10735-022-10108-3 36344754

[69] Hattori A (2017). Cancer progression by reprogrammed BCAA metabolism in myeloid leukaemia. Nature.

[70] Shu, X. *et al.* BCAT1 Activates PI3K/AKT/mTOR pathway and contributes to the angiogenesis and tumorigenicity of gastric cancer. *Frontiers in Cell and Developmental Biology* 1345 (2021). 10.3389/fcell.2021.659260 PMC8215359 34164393

[71] Xu Y (2018). Overexpression of BCAT1 is a prognostic marker in gastric cancer. Hum. Pathol..

[72] Wang P (2018). BCAT1 promotes proliferation of endometrial cancer cells through reprogrammed BCAA metabolism. Int. J. Clin. Exp. Pathol..

[73] Lin X, Tan S, Fu L, Dong Q (2020). BCAT1 overexpression promotes proliferation, invasion, and Wnt signaling in non-small cell lung cancers. Onco. Targets. Ther..

[74] Li G-S (2022). BCAT1: A risk factor in multiple cancers based on a pan-cancer analysis. Cancer Med..

[75] Yu M (2022). BCAT1 promotes lung adenocarcinoma progression through enhanced mitochondrial function and NF-$$\kappa$$B pathway activation. J. Zhejiang Univ.-Sci. B.

[76] van der Mijn JC (2022). Transcriptional and metabolic remodeling in clear cell renal cell carcinoma caused by ATF4 activation and the integrated stress response (ISR). Mol. Carcinog..

[77] Green NH (2019). MTHFD2 links RNA methylation to metabolic reprogramming in renal cell carcinoma. Oncogene.

[78] Noguchi K (2018). The mitochondrial one-carbon metabolic pathway is associated with patient survival in pancreatic cancer. Oncol. Lett..

[79] Huang J, Qin Y, Lin C, Huang X, Zhang F (2021). MTHFD2 facilitates breast cancer cell proliferation via the AKT signaling pathway. Exp. Ther. Med..

[80] Pikman Y (2016). Targeting MTHFD2 in acute myeloid leukemia. J. Exp. Med..

[81] Yu C (2020). Down-regulation of MTHFD2 inhibits NSCLC progression by suppressing cycle-related genes. J. Cell Mol. Med..

[82] Gao, Y., Feng, L., Zhang, L., Geng, J. & Zhang, E. ATF4/MYC regulates MTHFD2 to promote NSCLC progression by mediating redox homeostasis. *Dis. Markers*** 2022**, 7527996 (2022). 10.1155/2022/7527996 PMC9425107 36051358

[83] Liu X (2016). Methylenetetrahydrofolate dehydrogenase 2 overexpression is associated with tumor aggressiveness and poor prognosis in hepatocellular carcinoma. Dig. Liver Dis..

[84] Ju, H.-Q. *et al.* Modulation of redox homeostasis by inhibition of MTHFD2 in colorectal cancer: Mechanisms and therapeutic implications. *JNCI: J. Natl. Cancer Inst.* **111**, 584–596 (2019). 10.1093/jnci/djy160 PMC6579745 30534944

[85] Koufaris C (2016). Suppression of MTHFD2 in MCF-7 breast cancer cells increases glycolysis, dependency on exogenous glycine, and sensitivity to folate depletion. J. Proteome Res..

[86] Lee J (2021). A novel oral inhibitor for one-carbon metabolism and checkpoint kinase 1 inhibitor as a rational combination treatment for breast cancer. Biochem. Biophys. Res. Commun..

[87] Li Q (2021). MTHFD2 promotes ovarian cancer growth and metastasis via activation of the STAT3 signaling pathway. FEBS Open Bio..

[88] Liu X (2021). Non-metabolic function of MTHFD2 activates CDK2 in bladder cancer. Cancer Sci..

[89] Zhu Z (2022). Folate enzyme MTHFD2 links one-carbon metabolism to unfolded protein response in glioblastoma. Cancer Lett..

[90] Wu S (2022). Knockdown of MTHFD2 inhibits proliferation and migration of nasopharyngeal carcinoma cells through the ERK signaling pathway. Biochem. Biophys. Res. Commun..

[91] Wang, W. *et al.* The emerging role of MTHFD family genes in regulating the tumor immunity of oral squamous cell carcinoma. *J. Oncol.* **2022** (2022). 10.1155/2022/4867730 PMC9187492 35693982

[92] Nilsson R (2014). Metabolic enzyme expression highlights a key role for MTHFD2 and the mitochondrial folate pathway in cancer. Nat. Commun..

[93] Shang M (2021). The folate cycle enzyme MTHFD2 induces cancer immune evasion through PD-L1 up-regulation. Nat. Commun..

[94] Choudhury Y (2012). Attenuated adenosine-to-inosine editing of microRNA-376a* promotes invasiveness of glioblastoma cells. J. Clin. Investig..

[95] Gong J (2014). Comprehensive analysis of human small RNA sequencing data provides insights into expression profiles and miRNA editing. RNA Biol..

[96] Bo X (2018). The regulation and function of microRNA-377/RASSF8 signaling axis in gastric cancer. Oncol. Lett..

[97] He C, Wang L, Zhang J, Xu H (2017). Hypoxia-inducible microRNA-224 promotes the cell growth, migration and invasion by directly targeting RASSF8 in gastric cancer. Mol. Cancer.

[98] Chen Y, Bian L, Zhang Y (2018). MiR-505 mediates methotrexate resistance in colorectal cancer by targeting RASSF8. J. Pharm. Pharmacol..

[99] Zhang X (2022). RASSF8-AS1 displays low expression in colorectal cancer and up-regulates RASSF8 to suppress cell invasion and migration. Pathol.-Res. Pract..

[100] Huang Y (2016). Over-expressed miR-224 promotes the progression of cervical cancer via targeting RASSF8. PLoS ONE.

[101] Li, X. *et al.* CircCERS6 suppresses the development of epithelial ovarian cancer through mediating miR-630/RASSF8. *Biochem. Genet.* 1–19 (2022). 10.1007/s10528-022-10227-2 35676548

[102] Wang J (2015). RASSF8 regulates progression of cutaneous melanoma through nuclear factor-$$\kappa$$b. Oncotarget.

[103] Zhang L (2015). RASSF8 downregulation promotes lymphangiogenesis and metastasis in esophageal squamous cell carcinoma. Oncotarget.

[104] Falvella F (2006). Identification of RASSF8 as a candidate lung tumor suppressor gene. Oncogene.

[105] Wang L, Liu W, Zhang Y, Huang X (2017). The miR-224 promotes non-small cell lung cancer cell proliferation by directly targeting RASSF8. Eur. Rev. Med. Pharmacol. Sci..

[106] Wang, T. *et al.* MiR-505-5p inhibits proliferation and promotes apoptosis of osteosarcoma cells via regulating RASSF8 expression. *J. BU ON. Off. J. Balkan Union Oncol.* **26**, 599–605 (2021). 34077011

[107] Kajdasz A (2021). Identification of RCC subtype-specific microRNAs-meta-analysis of high-throughput RCC tumor microRNA expression data. Cancers.

[108] Beuselinck B (2015). Molecular subtypes of clear cell renal cell carcinoma are associated with sunitinib response in the metastatic setting. Clin. Cancer Res..

[109] Andrews, S. *et al.* FastQC: A quality control tool for high throughput sequence data (2010).

[110] Rosenbloom KR (2015). The UCSC genome browser database: 2015 update. Nucleic Acids Res..

[111] Kozomara A, Griffiths-Jones S (2014). miRBase: Annotating high confidence microRNAs using deep sequencing data. Nucleic Acids Res..

[112] Ye J, McGinnis S, Madden TL (2006). BLAST: Improvements for better sequence analysis. Nucleic Acids Res..

[113] Langmead B, Trapnell C, Pop M, Salzberg SL (2009). Ultrafast and memory-efficient alignment of short DNA sequences to the human genome. Genome Biol..

[114] Benjamini Y, Hochberg Y (1995). Controlling the false discovery rate: A practical and powerful approach to multiple testing. J. Roy. Stat. Soc.: Ser. B (Methodol.).

[115] Kiran A, Baranov PV (2010). DARNED: A DAtabase of RNa EDiting in humans. Bioinformatics.

[116] Ramaswami G, Li JB (2014). RADAR: A rigorously annotated database of A-to-I RNA editing. Nucleic Acids Res..

[117] Peng Z (2012). Comprehensive analysis of RNA-Seq data reveals extensive RNA editing in a human transcriptome. Nat. Biotechnol..

[118] Nigita G (2018). Tissue and exosomal miRNA editing in non-small cell lung cancer. Sci. Rep..

[119] Smigielski EM, Sirotkin K, Ward M, Sherry ST (2000). dbSNP: A database of single nucleotide polymorphisms. Nucleic Acids Res..

[120] Love MI, Huber W, Anders S (2014). Moderated estimation of fold change and dispersion for RNA-seq data with DESeq2. Genome Biol..

[121] Memczak S (2013). Circular RNAs are a large class of animal RNAs with regulatory potency. Nature.

[122] Zhao Q (2020). Transcriptomic characterization and innovative molecular classification of clear cell renal cell carcinoma in the chinese population. Cancer Cell Int..

[123] Robinson MD, McCarthy DJ, Smyth GK (2010). edgeR: A Bioconductor package for differential expression analysis of digital gene expression data. Bioinformatics.

[124] Xie C (2011). KOBAS 2.0: A web server for annotation and identification of enriched pathways and diseases. Nucleic Acids Res..

