# Supplementary material for: Identification of microRNA editing sites in clear cell renal cell carcinoma
Source: Sci Rep. 2023 Sep 13;13:15117. doi: 10.1038/s41598-023-42302-y (PMC10499803; doi:10.1038/s41598-023-42302-y)
Supplement: Supplementary file 1 — Supplementary Information 1. [file 41598_2023_42302_MOESM1_ESM.docx]

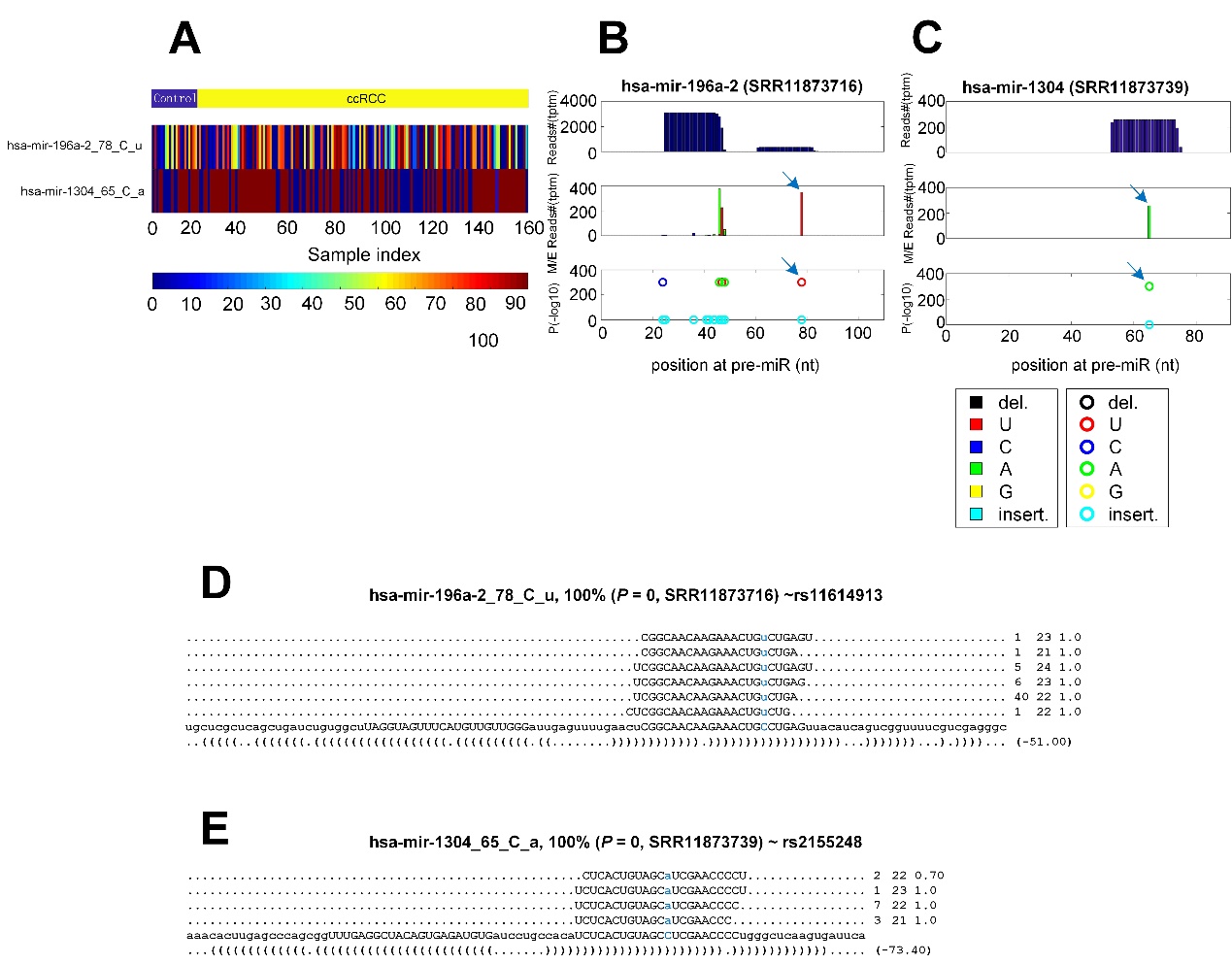


**Supplementary Figure S1**. The details of two SNPs in miRNAs identified from the ccRCC samples selected. (A) The editing levels of two SNPs in the 176 selected sRNA-seq profiles. (B) The MiRME map of hsa-mir-196a-2 in one of the normal samples selected (SRR11873716). (C) The MiRME map of hsa-mir-1304 in one of the ccRCC samples selected (SRR11873739). (D) The details of hsa-mir-196a-2_78_C_u in ERR4367258. (E) The details of hsa-mir-1304_65_C_a in ERR4367258.


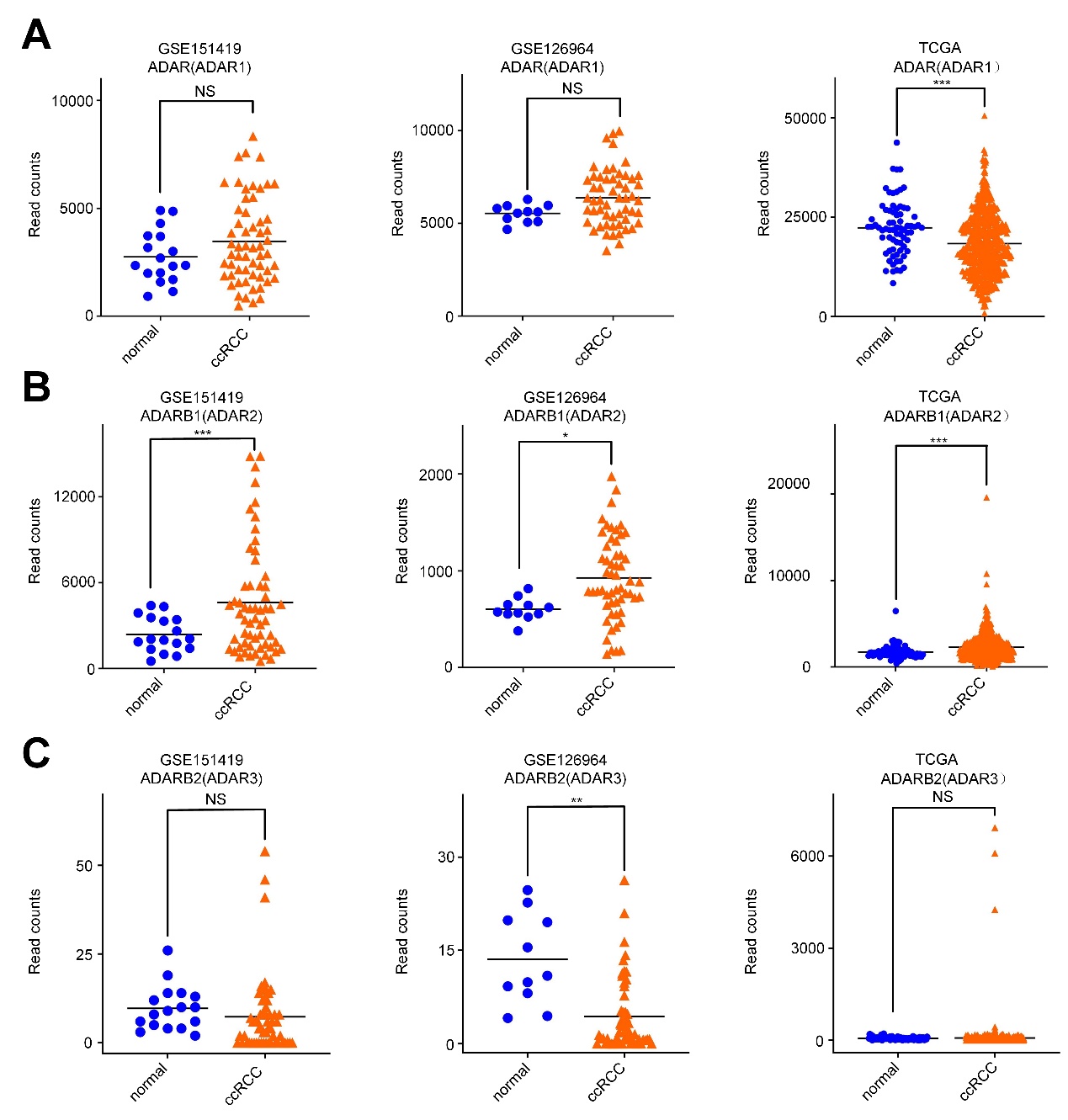


**Supplementary Figure S2.** Comparisons of expression levels of the ADAR genes in three sets of gene expression profiles of ccRCC. The edgeR package was used to compare the expression levels of ADARs. In all parts, *: *p* < 0.05; **: *p* < 0.01, ***: *p* < 0.001, and NS: not significant, i.e., *p* ≥ 0.05. (A) Comparison of expression level of ADAR1 in the three sets of gene expression datasets. (B) Comparison of expression level of ADAR2 in the three sets of gene expression datasets. (C) Comparison of expression level of ADAR3 in the three sets of gene expression datasets.

**
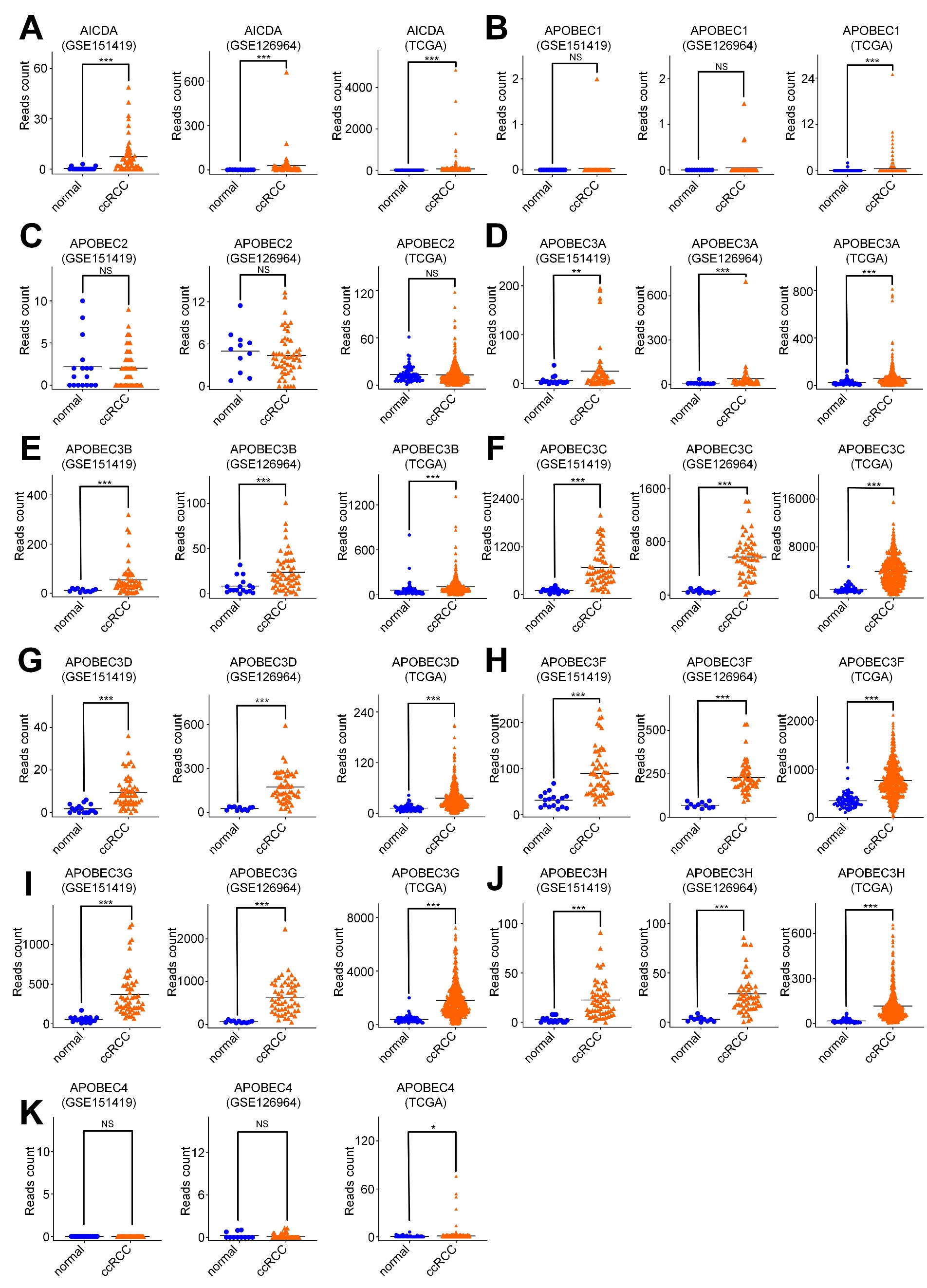
**

**Supplementary Figure S3**. Comparisons of expression levels of the APOBEC genes in three sets of gene expression profiles of ccRCC. The edgeR package was used to compare the expression levels of ADARs. In all parts, *: *p* < 0.05; **: *p* < 0.01, ***: *p* < 0.001, and NS: not significant, i.e., *p* ≥ 0.05. (A) Comparison of expression level of AICDA in the three sets of gene expression datasets. (B) Comparison of expression level of APOBEC1 in the three sets of gene expression datasets. (C) Comparison of expression level of APOBEC2 in the three sets of gene expression datasets. (D) Comparison of expression level of APOBEC3A in the three sets of gene expression datasets. (E) Comparison of expression level of APOBEC3B in the three sets of gene expression datasets. (F) Comparison of expression level of APOBEC3C in the three sets of gene expression datasets. (G) Comparison of expression level of APOBEC3D in the three sets of gene expression datasets. (H) Comparison of expression level of APOBEC3F in the three sets of gene expression datasets. (I) Comparison of expression level of APOBEC3G in the three sets of gene expression datasets. (J) Comparison of expression level of APOBEC3H in the three sets of gene expression datasets. (K) Comparison of expression level of APOBEC4 in the three sets of gene expression datasets.


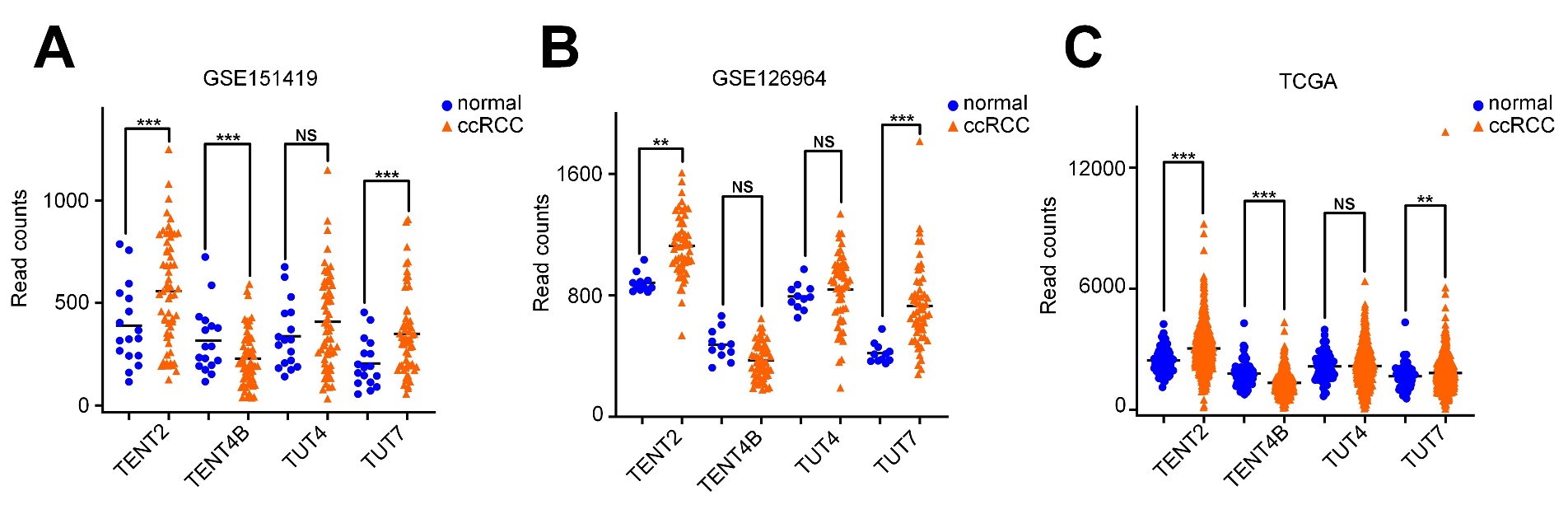


**Supplementary Figure S4**. Comparisons of expression levels of the TENT genes in three sets of gene expression profiles of ccRCC. The edgeR package was used to compare the expression levels of ADARs. In all parts, *: *p* < 0.05; **: *p* < 0.01, ***: *p* < 0.001, and NS: not significant, i.e., *p* ≥ 0.05. (A) Comparisons of expression levels of TENT2, TENT4B, TUT4 and TUT7 in one set (GSE151419) of the ccRCC gene expression datasets selected. (B) Comparisons of expression levels of TENT2, TENT4B, TUT4 and TUT7 in one set (GSE126964) of the ccRCC gene expression datasets selected. (C) Comparisons of expression levels of TENT2, TENT4B, TUT4 and TUT7 in one set (TCGA) of the ccRCC gene expression datasets selected.

**
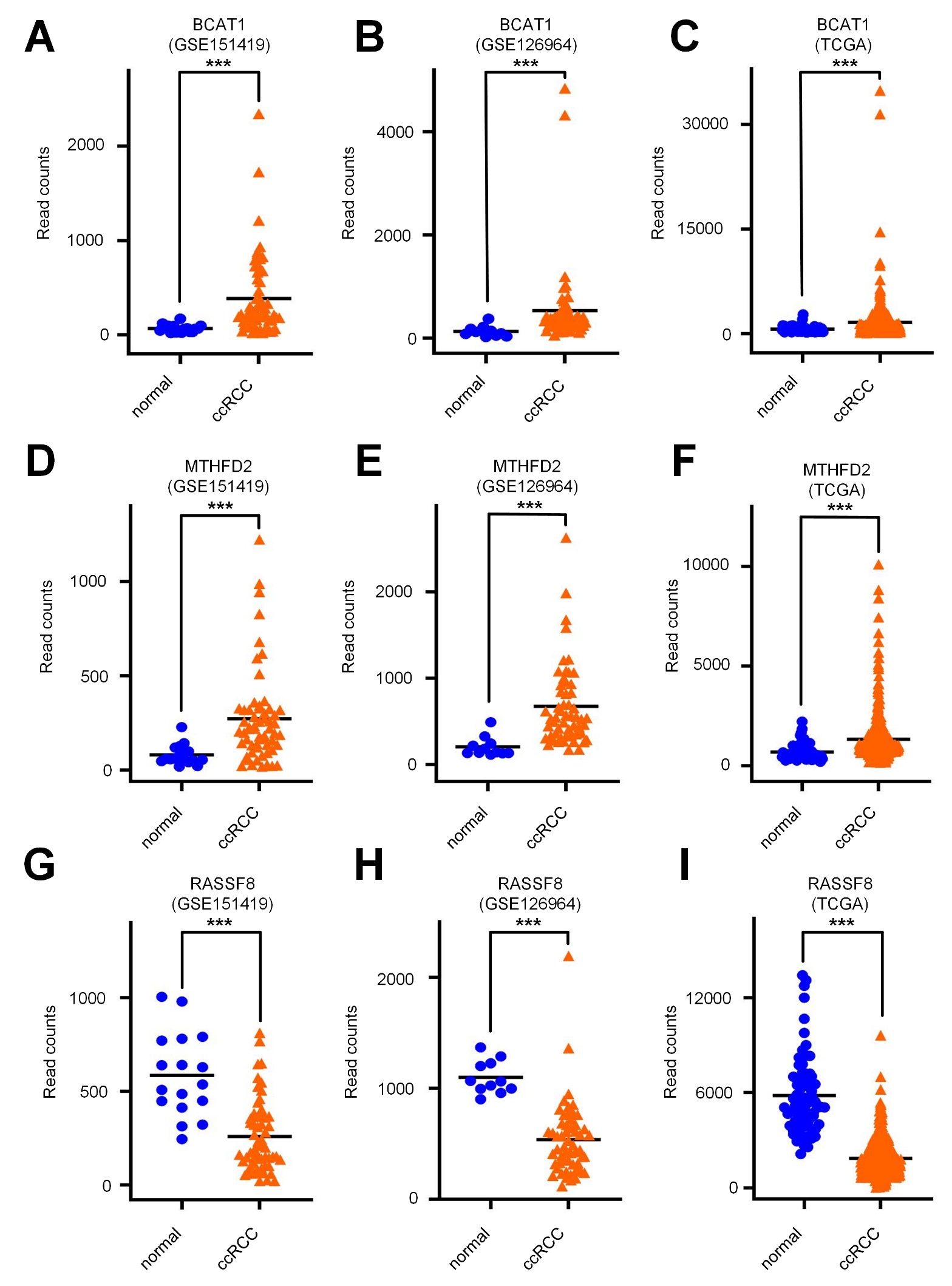
**

**Supplementary Figure S5**. Comparisons of expression levels of BCAT1, MTHFD2 and RASSF8 in three sets of gene expression profiles of ccRCC. (A-C) Comparison of expression level of BCAT1 in the three sets of gene expression datasets. (D-F) Comparison of expression level of MTHFD2 in the three sets of gene expression datasets. (G-I) Comparison of expression level of RASSF8 in the three sets of gene expression datasets.

**
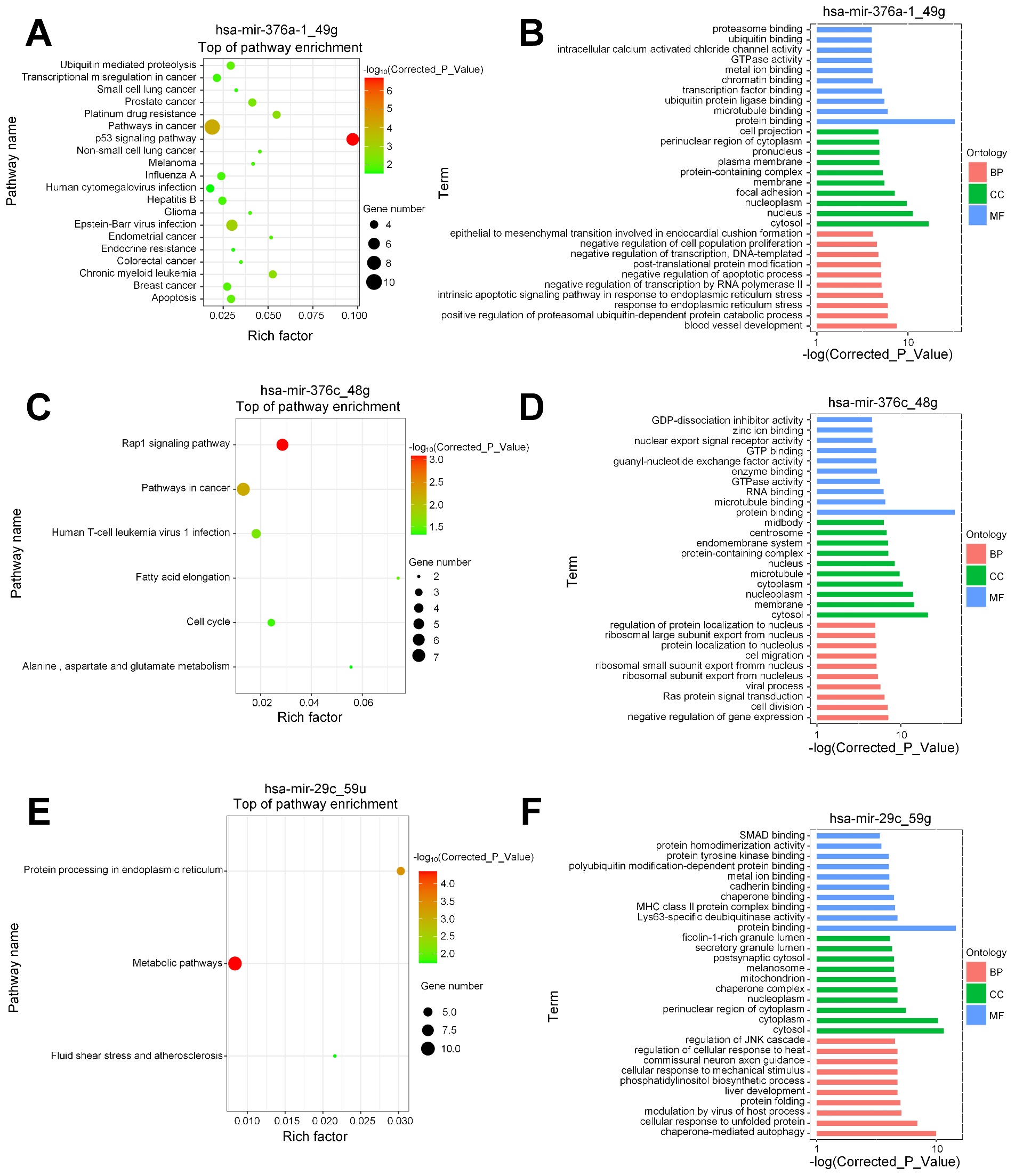
**

**Supplementary Figure S6**. The enriched KEGG pathways and GO terms of selected new targets of hsa-mir-376a-1_49g, hsa-mir-376c_48g, and hsa-mir-29c_59u. (A, C, E) The enriched KEGG pathways of the new target genes of hsa-mir-376a-1_49g, hsa-mir-376c_48g and hsa-mir-29c_59u, respectively. (B, D, F) The ten most significant GO terms of the new target genes of hsa-mir-376a-1_49g, hsa-mir-376c_48g and hsa-mir-29c_59u. The GO terms with the smallest corrected *p*-values in each of three major GO categories, i.e., Molecular Function (MF), Cellular Component (CC) and Biological Process (BP), were shown respectively.
